# Contribution of DNA adenine methylation to gene expression heterogeneity in *Salmonella enterica*

**DOI:** 10.1093/nar/gkaa730

**Published:** 2020-09-21

**Authors:** María A Sánchez-Romero, David R Olivenza, Gabriel Gutiérrez, Josep Casadesús

**Affiliations:** Departamento de Genética, Facultad de Biología, Universidad de Sevilla, Apartado 1095, Seville 41080, Spain; Departamento de Genética, Facultad de Biología, Universidad de Sevilla, Apartado 1095, Seville 41080, Spain; Departamento de Genética, Facultad de Biología, Universidad de Sevilla, Apartado 1095, Seville 41080, Spain; Departamento de Genética, Facultad de Biología, Universidad de Sevilla, Apartado 1095, Seville 41080, Spain

## Abstract

Expression of *Salmonella enterica* loci harboring undermethylated GATC sites at promoters or regulatory regions was monitored by single cell analysis. Cell-to-cell differences in expression were detected in ten such loci (*carA*, *dgoR, holA, nanA*, *ssaN, STM1290*, *STM3276, STM5308, gtr* and *opvAB*), with concomitant formation of ON and OFF subpopulations. The ON and OFF subpopulation sizes varied depending on the growth conditions, suggesting that the population structure can be modulated by environmental control. All the loci under study except *STM5308* displayed altered patterns of expression in strains lacking or overproducing Dam methylase, thereby confirming control by Dam methylation. Bioinformatic analysis identified potential binding sites for transcription factors OxyR, CRP and Fur, and analysis of expression in mutant backgrounds confirmed transcriptional control by one or more of such factors. Surveys of gene expression in pairwise combinations of Dam methylation-dependent loci revealed independent switching, thus predicting the formation of a high number of cell variants. This study expands the list of *S. enterica* loci under transcriptional control by Dam methylation, and underscores the relevance of the DNA adenine methylome as a source of phenotypic heterogeneity.

## INTRODUCTION

Non mutational variation plays multiple roles in bacterial populations. For instance, formation of cell types with different surface antigens permits evasion of the immune system inside animal hosts (1). In biofilms, cell-to-cell variation can foster division of labor (2). Phenotypic heterogeneity can also facilitate survival in the presence of antibacterial agents (3,4) and of bacteriophages (5,6). The benefits of phenotypic variation remain hypothetical in other cases, mainly due to experimental difficulties. However, game theory analysis supports the view that cell-to-cell variation has adaptive value for bacterial populations, especially in variable and hostile environments (7,8). The variety of mechanisms that produce cell diversification, which is indicative of independent origin, provides additional, indirect evidence that phenotypic variation may have evolved by natural selection (9–11).

A widespread mechanism for formation of phenotypic variants in a population of isogenic bacterial cells is epigenetic control by DNA methylation (12). For instance, certain DNA methyltransferases belonging to restriction-modification systems undergo reversible switching between active and inactive states, thus generating cells in which the genome is methylated and cells in which the genome is nonmethylated (13–15). If the methylation state of promoters or regulatory regions affects transcription, each cell lineage displays a distinct transcriptomic profile. In turn, transcriptome differences can cause phenotypic differences, and the literature contains an increasing list of examples in which such differences affect virulence of bacterial pathogens (13,14,16).

Formation of bacterial lineages under DNA methylation control is also found in bacterial species that harbor DNA methyltransferases that are not part of restriction-modification systems (11). Paradigms of this class are the Dam methylase of gamma-proteobacteria and the CcrM methylase of alpha-proteobacteria, which methylate the adenosine moiety in 5′GATC3′ and 5′GANTC3′ motifs, respectively (17–19). In *Escherichia coli* and *Salmonella enterica*, most GATC sites are fully methylated except for a short period of hemimethylation after passage of the DNA replication fork (17,18,20). Certain GATC sites, however, can remain stably undermethylated (hemimethylated or nonmethylated) if the activity of Dam methylase is prevented by binding of proteins to cognate DNA sites (12,18). Hindrance of Dam methylase activity is favored at GATC sites flanked by nucleotide sequences that decrease Dam processivity (21), and nonmethylation occurs when DNA methylase activity is blocked during two consecutive DNA replication rounds (22).

Combinations of methylated and undermethylated GATCs at regulatory regions upstream of promoters can regulate transcription in a bistable manner, giving rise to OFF and ON cells (22). In such systems, the existence of two or more binding sites for a transcription factor permits alternative binding patterns, one of which activates transcription. A paradigm of bistable expression regulated by Dam methylation is *pap*, a fimbrial operon of uropathogenic *E. coli* under transcriptional control the leucine-responsive regulatory protein, Lrp (23–25). Alternative patterns of Lrp binding and GATC undermethylation generate the OFF and ON states, which give rise to nonfimbriated and fimbriated subpopulations (23). Additional examples of formation of DNA methylation patterns involve the transcriptional regulators OxyR, Fur and HdfR (26–31). The number of GATC sites involved and the architecture of the regulatory region is different in each case but all have in common the formation of methylated and undermethylated GATCs (12).

Undermethylation of specific GATC sites can be detected by genome digestion with restriction enzymes followed by deep sequencing (32,33). An alternative procedure is whole genome analysis by single molecule real-time sequencing (SMRT), a technology that permits detection of *N*^6^-methyl-adenine at a single base resolution (34). In this study, we have used previously published SMRT sequencing data (28) to assemble a catalog of undermethylated GATC sites in the genome of *S. enterica* ser. Typhimurium strain ATCC 14028. Single cell analysis using fluorescent reporter proteins GFP, mCherry and mOrange reveals that a fraction of such loci undergo heterogeneous expression under Dam methylation control, with concomitant formation of cells in ON and OFF transcriptional states. We thus identify novel loci with heterogeneous expression controlled by Dam methylation in *S. enterica*. We also show that seven such loci undergo ON/OFF switching in an independent manner, an observation that outlines the relevance of the DNA adenine methylome as a powerhouse for the formation of phenotypic variants.

## MATERIALS AND METHODS

### Bacterial strains, bacteriophages and strain construction

The strains of *S. enterica* used in this study belong to serovar Typhimurium and derive from strain ATCC 14028 (Supplementary Table S1). For simplicity, *S. enterica* serovar Typhimurium is named *S. enterica* throughout the text. Plasmid pTP166 (35) was kindly provided by Martin G. Marinus, University of Massachusetts, Worcester, MA. For construction of *gfp* transcriptional fusions, a fragment containing the promoterless green fluorescent protein (*gfp*) gene and the chloramphenicol resistance cassette was amplified from pZEP07 (36) using primers listed in Supplementary Table S2. The constructs were integrated into the chromosome of *S. enterica* using the lambda Red recombination system (37). For construction of strains harboring mCherry transcriptional fusions, a DNA fragment containing the promoterless *mCherry* gene and the kanamycin resistance cassette was PCR-amplified from pDOC-R, an *mCherry*-containing derivative of plasmid pDOC (a gift from Steve Busby's lab, University of Birmingham, UK). For construction of strains harboring mOrange transcriptional fusions, the *mOrange2* gene and the kanamycin resistance cassette were PCR-amplified from pGEMT-mOrange (kindly provided by Carmen R. Beuzón, University of Málaga, Spain). Transductional crosses using phage P22 HT 105/1 [(38) and G. Roberts, unpublished] were used for construction of strains lacking individual transcription factors (OxyR, CRP, Lrp or Fur). Strains carrying pairwise combinations of GFP and either mCherry or mOrange fusions were also constructed by P22 HT transduction. The transduction protocol has been described elsewhere (39). To obtain phage-free isolates, transductants were purified by streaking on green plates (40).

### Media and culture conditions

Bertani's lysogeny broth (LB) contained tryptone 10 g/l, yeast extract 5 g/l and NaCl 5 g/l. The composition of ISM (intracellular salts medium) was 5 mM KCl, 7.5 mM (NH_4_)_2_SO_4_, 0.5 mM K_2_SO_4_, 38 mM glicerol (0.3% v/v), 0.1% casamino acids, 8 μM MgCl_2_, 337 μM H_3_PO_4_, 80 mM MES (for titration to pH 5.8). Solid LB contained agar at 1.5% final concentration. All cultures were grown at 37°C. Oxygen limitation was achieved by growth without shaking in borosilicate tubes containining 5 ml of LB. To grow *oxyR* mutants on LB agar, 75 μl of a 10 mg/ml catalase solution (Sigma-Aldrich, St Louis, MO, USA) were spread on the surface of the plates.

### Analysis of SMRT sequencing data

Bioinformatic analysis was performed on raw SMRT sequencing data described in a previous study (28). The Pacific Biosciences’ SMRT Portal platform, v. 2.1.0 was used to identify modQVs at each position. These values were computed as the −10 log (*P*-value) based on the distributions of the kinetics of interpulse durations (IPD ratios). A modQV score of 20 is the minimum default threshold and corresponds to a *P*-value of 0.01 (28). All the undermethylated positions detected in this work are under this threshold.

### Identification of undermethylated GATC sites in the genome of *Salmonella enterica* Serovar Typhimurium ATCC 14028

A homemade Perl script was used to identify undermethylated GATC sites among the 19 229 GATCs present in the *S. enterica* ATCC 14028 genome. Another in-house Perl script identified pairs or higher order clusters of GATC motifs separated by <256 nucleotides (41). The Kolmogorov–Smirnov test for two samples, implemented in PAST (42), was used to test whether the distribution of undermethylated GATCs in the *S. enterica* genome was homogeneous or heterogeneous.

### Identification of DNA sequence motifs for binding of transcription factors

The FIMO tool from the MEME software suite (43) with default settings was used to scan a sequence database set of DNA sequences from nine loci (*carA, dgoR, holA, nanA, ssaN, STM1290, STM3276, gtr* and *opvAB*) containing 600 bp upstream and 100 pb downstream of the transcription start site. Individual matches to OxyR, CRP and Fur binding motifs were sought.

### Flow cytometry

Flow cytometry was used to monitor expression of transcriptional GFP fusions. Data acquisition was performed using a Cytomics FC500-MPL cytometer (Beckman Coulter, Brea, CA, USA) and data were analyzed with FlowJo X version 10.0.7r software (Tree Star, Inc., Ashland, OR). *S. enterica* cultures were washed and re-suspended in phosphate-buffered saline (PBS) for fluorescence measurement. Fluorescence values for 100 000 events were compared with the data from the reporter-less control strain, thus yielding the fraction of ON and OFF cells.

### Fluorescence microscopy

Strains containing individual or pairwise combinations of GFP and mCherry/mOrange fusions were grown at 37°C. Samples of 1.5 ml were collected by centrifugation at 3400 × g for 5 min. Cells were placed on an agarose slab (0.9% agarose/1% LB medium) pre-warmed at 37°C. Images were captured with a Zeiss Apotome fluorescence microscope equipped with a 100× Plan Apochromat objective and an incubation system that allows cultivation and observation of living cells. Pictures were taken using an Axiocam 506 camera, and the images were analyzed using ImageJ software (Wayne Rasband, Research Services Branch, National Institute of Mental Health, MD, USA).

## RESULTS

### Distribution of undermethylated GATC sites in the *Salmonella enterica* genome

The methylation state of GATC sites in the chromosome of *S. enterica* strain ATCC 14028 grown in LB was inferred from raw SMRT data obtained in a previous study (28). Bioinformatic analysis revealed the presence of 46 undermethylated GATC sites. Among such sites, 20 were hemimethylated, and 13 were nonmethylated (Supplementary Table S3). A Kolmogorov–Smirnov test for uniform distribution indicated that the 33 undermethylated GATCs were not randomly distributed in the *S. enterica* genome. A list of the 46 nonmethylated sites detected (20 in hemimethylated GATCs and 26 (13 × 2) in nonmethylated GATCs is presented in Supplementary Table S3, indicating the DNA strand and the genome location. Most undermethylated sites (42/46) were located in clusters that included 2, 3, or 4 GATC sites at distances below the 1/256 distance predicted for random occurrence (41). Table 1 includes only the 16 loci containing GATC sites at promoters or regulatory regions (known or putative), and indicates their methylation state. The distribution of these sites was as follows: one undermethylated GATC in the *dgoR* gene; two in *carA*, *ssaN*, *slrA*, *STM1290* and *yihU*; three in *STM5047*, *STM5308*, *STM3726* and *STM2047*; four in *ftnB, gtr, opvAB* and *STM4889*; five in *holA*; and eight in *nanA*. The list includes undermethylated GATC sites previously shown to be involved in transcriptional control of the *gtr and opvAB operons* (28,29). This observation served as an internal control, indicating that the bioinformatic analysis was reliable.

**Table 1.** Loci harboring undermethylated GATCs in regulatory regions

Locus	Gene product	Number of GATCs	Number of undermethylated GATCs	Position(s) of GATC undermethylated A(s)^a^	Methylation state^b^
*carA*	Carbamoyl-phosphate synthase small chain	2	2	−511, −206	N, H
*dgoR*	Galactonate operon transcriptional repressor	1	1	−147	H
*ftnB*	Ferritin-like protein	4	2	−340, −156	H, H
*gtr*	O-antigen glycotransferase	4	2	−69, −56	N, N
*holA*	DNA polymerase III, delta subunit	5	1	+4	H
*nanA*	N-acetylneuraminate lyase	8	1	−58	H
*opvAB*	O-antigen chain length regulation	4	2	−178, −105	N, N
*slrA*	Glucitol/sorbitol-specific enzyme IIC component	2	1	−86	H
*ssaN*	Type III secretion ATP synthase	2	1	−202	N
*STM1290*	N-acetylmannosamine-6-phosphate-2-epimerase	2	1	−465	N
*STM2047*	Hypothetical protein	3	1	−68	H
*STM3726*	Putative mannitol dehydrogenase	3	1	−68	H
*STM4889*	Putative Na^+^/galactosidase symporter	4	1	−171	N
*STM5047*	Putative cytoplasmic protein	3	1	−108	H
*STM5308*	Sugar transporter	3	1	−66	N
*yihU*	Hypothetical oxidoreductase	2	1	−66	N

^a^Relative to known or predicted transcription start sites.

^b^N: nonmethylated; H: hemimethylated.

### Expression patterns of loci harboring undermethylated GATC sites in putative regulatory regions

**Figure 1.**
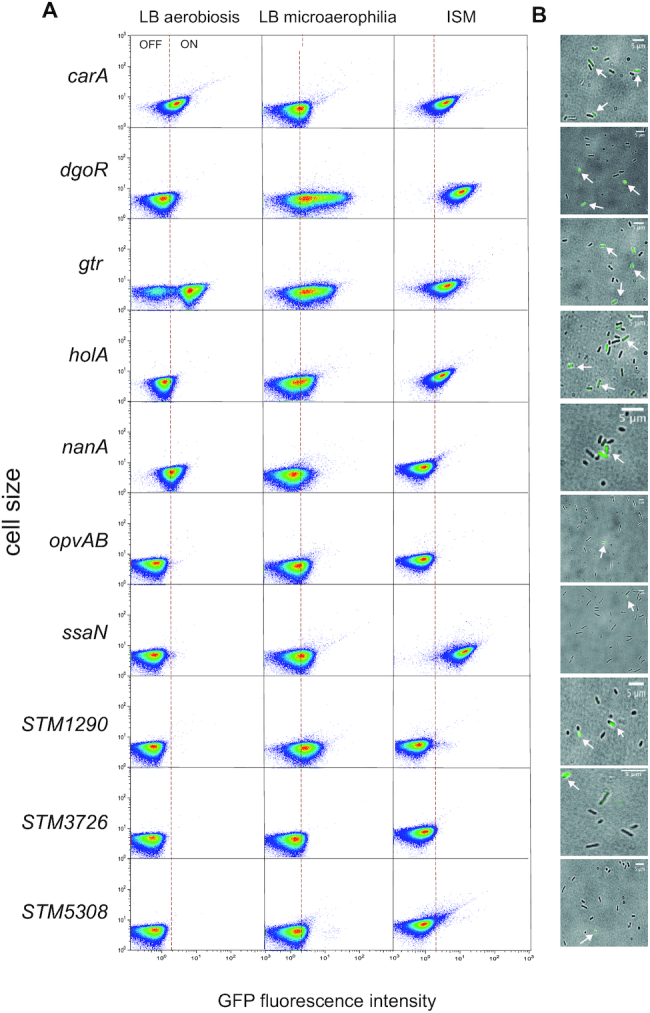
Expression of loci harboring undermethylated GATC sites at putative promoters or regulatory regions, monitored by single cell analysis. (**A**) Flow cytometry analysis of strains carrying *gfp* transcriptional fusions downstream of the loci under study. The GFP fluorescence intensity distribution was examined in cultures grown in LB under aerobiosis, LB under miare OKcroaerophilia and intracellular salts medium (ISM). Dot plots represent the forward scatter (cell size) versus fluorescence intensity. (**B**) Visualization of *Salmonella* cells by fluorescence microscopy. For each locus, the image shown was obtained under the growth condition in which bistability was most conspicuous (LB under microaerophilia for *carA*, *dgoR*, *gtr*, *holA*, *STM1290*, *STM3276* and *STM5308*, and LB under aerobiosis for *opvAB* and *ssaN*). White arrows signalize ON cells. Scale bars indicate 5 μm.

The expression patterns of the 16 loci harboring undermethylated GATC sites potentially involved in transcriptional control (*dgoR*, *carA*, *ssaN*, *slrA, yihU, STM1290*, *STM5047*, *STM5308*, *STM3726*, *STM2047*, *nanA*, *ftnB opvAB*, *gtr*, *holA* and *STM4889*) were investigated using transcriptional fusions with the green fluorescent protein (GFP) gene. Each fusion was constructed downstream of the locus under study, and expression was monitored by flow cytometry. Cultures were grown in LB under aerobiosis, LB under microaerophilia and intracellular salts medium (ISM). These choices took into consideration the *Salmonella* lifestyle: LB under microaerophilia permits reductionist imitation of the intestinal environment (44) while ISM mimics the intracellular environment (45). Expression of six loci (*ftnB*, *slrA*, *yihU*, *STM2047, STM4889* and S*TM5047*) was low or absent under every growth condition (Supplementary Figure S1), and the loci were excluded from further analysis. GFP fusions in the remaining 10 loci were found to be active, and a remarkable observation was that expression appeared to be heterogeneous in one or more culture conditions, with formation of cells in OFF and ON states (Figure 1, panel A). Visualization of individual *S. enterica* cells by fluorescence microscopy confirmed the existence of ON and OFF cells (Figure 1, panel B).

For certain loci, different proportions of OFF and ON cells were found depending on the growth conditions, indicating that expression of such loci is susceptible to environmental influence. For instance, the OFF subpopulation found in LB under microaerophilia for *dgoR*, *carA* and *nanA* decreased or even disappeared in ISM and in LB under aerobiosis (Figure 1). Differences in subpopulation sizes depending on growth conditions were also detected for *ssaN*, *holA* and *STM1290* (Figure 1).

### Role of Dam methylation in transcriptional control of undermethylated loci

**Figure 2.**
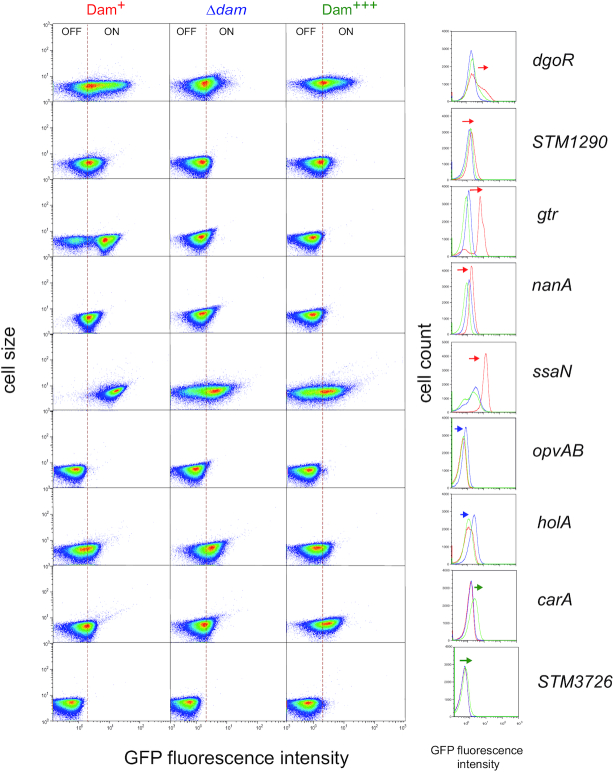
Single cell analysis of Dam methylation-dependent control of *carA, dgoR*, *gtr*, *holA*, *nanA*, *opvAB*, *ssaN*, *STM1290* and *STM3726*. Gene expression was measured by flow cytometry in the wild type (left column and red line in the histograms), in a *dam* mutant (middle column and blue line in the histograms) and under Dam methylase overproduction (right column and green line in the histograms). Dot plots represent the forward scatter (cell size) versus fluorescence intensity. Histograms represent the frequency of cells with different GFP intensities, and changes in the pattern of expression are indicated with arrows. The growth condition shown for *dgoR*, *STM1290*, *holA* and *carA* is LB under microaerophilia; for *gtr*, *nanA*, *opvAB* and *STM3726* is LB under aerobiosis; and for *ssaN* is ISM minimal medium. More detailed information is provided in Supplementary Figure S2.

Loci showing heterogeneous expression (*carA*, *dgoR, gtrA, holA, nanA*, *opvAB, ssaN, STM1290*, *STM3276* and *STM5308*) were tested for Dam methylation control by monitoring the levels of GFP fluorescence in three genetic backgrounds: the wild type strain, a *dam* mutant, and a strain carrying the *dam* gene on the multicopy plasmid pTP144 (35). GFP fluorescence was measured by flow cytometry under the three growth conditions described above. Both heat maps and histograms are shown because each of these representations is more informative depending on the locus (Supplementary Figure S2). A simplified version of the same results is presented in Figure 2, which shows the expression pattern of each locus under the growth conditions that revealed Dam methylation-dependent control in a most conspicuous manner. *STM5308* is not included in Figure 2 because Dam-dependent regulation was not detected (see Supplementary Figure S2). The Dam methylation-dependent expression patterns observed for the remaining loci were as follows:

- In a *dam* background, the size of ON subpopulations decreased at four loci (*dgoR, STM1290, gtr* and *ssaN*), suggesting that GATC methylation is involved in transcriptional activation (Figure 2). In turn, a larger ON subpopulation was detected at two loci (*opvAB* and *holA*), suggesting Dam-dependent repression (Figure 2). Absence of Dam methylation had little effect, if any, on *carA, nanA* and *STM3726* expression (Figure 2). As above, *gtr* and *opvAB* served as internal controls as these loci have been previously shown to undergo transcriptional upregulation (*opvAB*) and transcriptional downregulation (*gtr*) in a *dam* background.
- Overexpression of Dam methylase increased the size of CarA^ON^ and STM3726^ON^ subpopulations, thereby indicating Dam-dependent activation of transcription (Figure 2).
- Detection of a smaller HolA^ON^ subpopulation upon Dam methylase overexpression confirmed Dam-dependent repression (Figure 2). At *nanA*, repression was especially severe, and Dam methylase overproduction abolished formation of the Nan^ON^ subpopulation (Figure 2).

### Identification of regulators involved in transcriptional control

**Figure 3.**
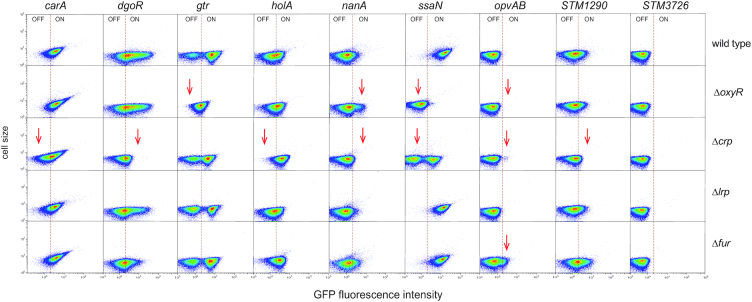
Flow cytometry analysis of *carA, dgoR*, *gtr*, *holA*, *nanA*, *opvAB*, *ssaN* and *STM1290* expression in the absence of individual transcriptional factors. Dot plots represent the forward scatter (cell size) versus fluorescence intensity. For *dgoR*, *holA, nanA, STM1290* and *STM3726*, the growth condition shown is LB under microaerophilia; for *gtr* and *opvAB* is LB under aerobiosis; and for *carA* and *ssaN* is ISM. More detailed information is provided in Supplementary Figure S3.

Undermethylation of GATC sites is usually a consequence of protein binding which hinders DNA methylase activity (11,12). The presence of undermethylated GATC sites upstream of genes (Table 1 and Supplementary Table S3) thus suggested that these sites might be located within or near binding sites for transcription factors. Because transcription factors OxyR, LRP, CRP and Fur have been previously shown to direct undermethylated GATC formation (12), expression of the loci under study was monitored in the absence of individual transcription factors. For this purpose, *gfp* fusions were transferred to *oxyR, lrp, crp* and *fur* mutants using P22 HT transduction. The resulting strains were grown, as above, in LB under aerobiosis, LB under microaerophilia and ISM medium, and the expression of the *gfp* fusions was monitored by flow cytometry. Strains harboring the same fusions in a wild type background were used as controls. The results from these experiments are shown in Supplementary Figure S3. A simplified version of the same results is presented in Figure 3, showing the expression pattern of each locus under the growth conditions in which altered expression is most easily seen. With the exception of *STM3726*, the remaining loci under study changed their expression pattern in one or more mutant backgrounds (Figure 3). However, none of the loci under study was found to be under Lrp control. Changes in the expression patterns of *gtr* and *opvAB* in an *oxyR* background are in agreement with the literature (28,29) thus serving as internal controls for the trials (Figure 3). Relevant observations can be summarized as follows:

- The subpopulations of CarA^OFF^, DgoR^OFF^, NanA^OFF^, SsaN^OFF^ and STM1290^OFF^ cells were larger in the absence of CRP, thereby suggesting transcriptional activation. In contrast, the size of the HolA^ON^ and OpvAB^ON^ subpopulations increased in a *crp* background, which may be indicative of repression by CRP. The ability of CRP to act either as a repressor or as an activator of transcription has been known for decades (46).
- In the absence of Fur, the size of the OpvAB^ON^ subpopulation increased, suggesting Fur-mediated repression. A Dam methylation-dependent locus repressed by Fur has been described in *E. coli* (27,47).
- In the absence of OxyR, the frequencies of SsaN^ON^ and OvpAB^ON^ cells decreased. The role of OxyR as an *opvAB* activator has been described previously (48).

To investigate whether the expression changes detected by flow cytometry might provide evidence for transcriptional control, potential DNA binding motifs for the transcription factors under study were sought at sequences flanking the GATCs. The FIMO tool from the MEME software suite was used for this purpose (49). Subsequently, Tomtom was used to compare the motifs found by MEME with known motifs contained at the Collect TF database, https://www.uniprot.org/database/DB-0198. Results from these searches are shown in Table 2 and Supplementary Table S4, and can be summarized as follows:

- Putative CRP binding sites were identified upstream of *carA*, *dgoR*, *nanA, ssaN* and *STM1290* (Table 2), in agreement with the observation that these loci are under positive control by CRP. In *dgoR* and *nanA*, GATCs where found within the putative CRP binding sites while in *carA*, *STM1290* and *ssaN*, GATC sites were found near (but not within) the putative CRP binding sequence. Note that ‘outsider’, nearby GATC sites can anyway be involved in transcriptional control, presumably as a consequence of the formation of higher order DNA structures in regions bound by transcription factors active as dimers or multimers (28).
- Putative OxyR binding sites were found in *gtr, opvAB*, and *ssaN*, which is consistent with genetic evidence indicating regulation by OxyR (Figure 3). For *gtr* and *opvAB*, regulation by OxyR is also in agreement with the literature (28,29,48).
- Putative binding sites for Fur were detected upstream of both *opvAB* and *gtr* (Table 2). Fur-mediated regulation was indeed detected for *opvAB* (Figure 3) but not for *gtr*, perhaps because the experimental conditions were not appropriate.
- Failure to identify binding sites for CRP upstream of *holA* (Table 2) might be indicative of indirect control.

**Table 2.** Identification of potential binding sites for transcription factors

Locus	Transcription factor involved	Predicted TF binding site (5′-3′)	Location relative to transcription start site
*carA*	CRP	CGATTGTAATTCTTATTACATTG	−499 to −477
*dgoR*	CRP	TTTTGTGATCTAAATTGTA	−154 to −132
*gtr*	CRP	TAACTTTAAACTATTGAATA	−100 to −82
	OxyR^b^	GATCGGTAACAACGATC	−134 to −119
	OxyR^b^	GATCGTTTATATCGATC	−70 to −55
	Fur	AATTAATAAGATAACAATAACTTTAAACTATTGAATA	−118 to −82
*holA*	CRP	Not found	
*nanA*	CRP	AAATGCGGTAGCGATCGCA	−145 to −127
	CRP	GTTTAGTGAAGCAGATCGCAC	−113 to −94
	OxyR	ATTGTGATTGTTGATGCCAT	−260 to −241
*opvAB*	CRP	AAAGTGTGCTCTGGTTTGCAA	−489 to −469
	OxyR^a^	ATAGTTTATATCTAT	−161 to −147
	OxyR^a^	GATCGATTTTAATTAT	−129 to −115
	OxyR^a^	ATAGTTTTTATCTAT	−88 to −74
	OxyR^a^	GATCGATATAACCAGT	−56 to −42
	Fur	CGTCACATAAAACAAAAACGATCAATTTTATTTATATGA	−199 to −161
*ssaN*	CRP	GTCTTATTTTCGGCACCTTAA	−435 to −415
	OxyR	CATTAATAAAATCGCTGAAACTTTGCAACGGCTTGT	−500 to −465
*STM1290*	CRP	ATGTGACCAAGCGCTAAAAAT	−428 to −408
	CRP	TGTGATGCATTTCATACTG	−283 to −265
	CRP	TATGACTGTGTTCACATTT	−102 to −84

^a^Sites described also by Cota *et al.* (28).

^b^Sites described also by Broadbent *et al.* (29).

In summary, in most cases genetic evidence for regulation by a given transcription factor was accompanied by the detection of a putative binding site for such a factor. Because our trials have been restricted to transcription factors previously known to be involved in Dam-dependent transcription (12), one cannot exclude that expression of the loci under study may be subjected to additional controls.

A notion well established in the literature is that GATC undermethylation is typically caused by DNA binding proteins (e.g. transcription factors) that hinder Dam methyltransferase activity (12,24,50). On this ground, we examined the methylation state of the *nanA* promoter in *crp^+^* and *crp^–^* backgrounds. Different methylation patterns were detected (Supplementary Figure S4), thus providing evidence that *nanA* GATC undermethylation is indeed a consequence of CRP binding. Methylation occlusion by transcription factors CRP, Fur and OxyR may likewise produce undermethylation at the other loci considered in this study.

### Tests of co-ordinated *vs* independent switching

**Figure 4.**
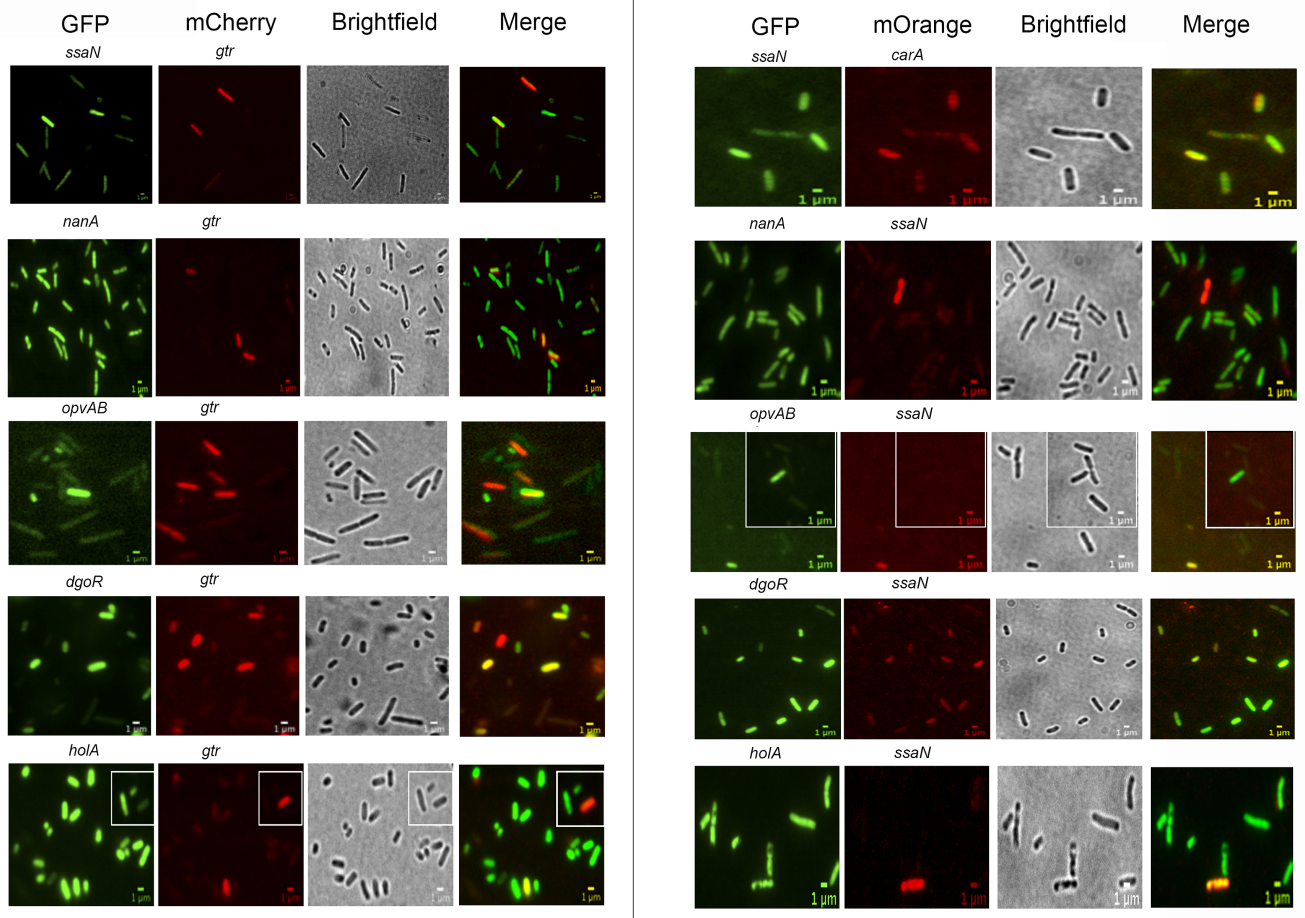
Tests of co-ordinated and/or independent switching of loci under transcriptional control by Dam methylation. Fluorescence images of *S. enterica* cells are shown, visualizing the expression of 10 pairwise combinations of gene fusions (*ssaN* versus *gtr, ssaN* versus *carA, nanA* versus *gtr, nanA* versus *ssaN, opvAB* versus *gtr, opvAB* versus *ssaN, dgoR* versus *gtrA, dgoR* versus *ssaN, holA* versus *gtr* and *holA* versus *ssaN)*. Each row shows a strain containing a GFP and either an mCherry or an mOrange transcriptional fusion. The growth condition shown for *ssaN* versus *gtr, nanA* versus *gtr, opvAB* versus *gt, ssaN* versus *carA, nanA* versus *ssaN, opvAB* versus *ssaN, dgoR* versus *ssaN* and *holA* versus *ssaN* is LB under aerobiosis; for *dgoR* versus *gtr*, and *holA* versus *gtr*, the growth condition shown is LB under microaerophilia. Scale bars indicate 1 μm.

The existence of locus-specific expression patterns and the variety of transcription factors involved raised the possibility that each locus might undergo independent switching. However, certain regulators appeared to control more than one Dam-methylation locus, thus suggesting the alternative possibility that co-ordinated regulation might occur. To address this issue, tests were performed to determine whether the loci under study undergo ON−OFF switching in a co-ordinated manner or independently. For this purpose, strains that carried pairwise combinations of a GFP fusion and either an mCherry or mOrange fusion were constructed, and the presence of OFF and ON cells was monitored by fluorescence microscopy. Altogether, 10 pairwise combinations were tested: *ssaN* versus *gtr, ssaN* versus *carA, nanA* versus *gtr, nanA* versus *ssaN, opvAB* versus *gtr, opvAB* versus *ssaN, dgoR* versus *gtrA, dgoR* versus *ssaN, holA* versus *gtr* and *holA* versus *ssaN*. Cells that expressed either mCherry/mOrange or GFP were detected in all trials, together with cells that expressed both fusions and cells that did not express mCherry/mOrange nor GFP. These observations provide evidence that the loci under examination are capable of independent switching (Figure 4).

## DISCUSSION

Heterogeneous gene expression in isogenic populations of cells can be the consequence of stochastic fluctuation of cell components, a phenomenon known as noise (51,52). In certain cases, cell-to-cell variations generated by noise can be propagated by feedback loops, giving rise to subpopulations of cells with distinct phenotypes (10,11,53). When two phenotypic lineages are formed, the phenomenon is known as bistability or bimodality (53,54). In bacterial populations, examples of noise-driven bistability include (among others) control of sporulation, activation of genetic competence, antibiotic resistance, biofilm formation and *myo*-inositol utilization (9,53,55).

Alongside bistable switches that combine random and deterministic components, bacteria can employ purely deterministic strategies to generate phenotypic lineages, and a well known mechanism is transcriptional control by DNA methylation (12). In gamma-proteobacteria, control of bistability by DNA adenine (Dam) methylation usually requires the formation of methylation patterns (combinations of methylated and undermethylated GATC sites) at promoters and/or nearby regions involved in transcriptional control. Undermethylation (stable hemimethylation or nonmethylation) of a GATC site occurs when Dam methyltransferase activity is hindered by a DNA-bound protein (12). In *E. coli* and *Salmonella*, the list of proteins that are known to cause undermethylation of GATC sites includes transcription factors Lrp, OxyR, Fur and HdfR (12).

In this study, we have used undermethylated GATC sites in the *S. enterica* genome as indicators of transcriptional control by Dam methylation. Indeed, among 16 loci containing undermethylated GATCs at promoters or regulatory regions (known or putative), nine showed distinct expression patterns depending on Dam methylase availability (Figure 2). Detection of undermethylated DNA sites upon SMRT sequencing may thus be considered a fairly productive approach, alternative or complementary to genetic and transcriptomic screens (56,57), to identify genes under DNA methylation control.

A remarkable trait shared by the nine Dam-dependent loci under study is bistable expression, with concomitant formation of cells in OFF and ON transcriptional states (Figure 1). Altered OFF and ON subpopulation sizes upon lack and/or overproduction of Dam methyltransferase confirmed that bistability is under Dam methylation control (Figure 2 and Supplementary Figure S2). An additional observation was that the ON/OFF ratios for the Dam-dependent loci under study varied depending on the medium and/or the growth conditions (Figure 2 and Supplementary Figure S2), in agreement with seminal studies describing conditions that affected the formation of undermethylated GATC sites in the *E. coli* chromosome (58,59).

Evidence for environmental influence, together with the fact that undermethylation is usually caused by protein binding (18,50), led us to search for *S. enterica* factors that might be involved in transcriptional control of the loci under study. Single cell analysis in mutant strains lacking individual transcription factors OxyR, CRP and Fur confirmed that subpopulation formation was under the control of one or more such factors (Figure 3 and Supplementary Figure S3). Detection of transcriptional control of *opvAB* and *gtr* by OxyR was confirmatory of published data (28,29,48), thus providing an internal control for the survey. Among the novel observations made, variations in the size of the OpvAB^ON^ lineage were detected in the absence of either CRP or Fur (Figure 3 and Supplementary Figure S3), suggesting that these regulators may contribute to transcriptional control of the *opvAB* operon. Because *opvAB* controls O-antigen chain length (48), CRP and/or Fur might perhaps skew ON/OFF switching under specific environmental circumstances. Environmental control of bistability may be also envisaged, again in a speculative manner, for CRP-mediated control of *dgoR, nanA* and perhaps *holA*. Dam methylation-dependent control of *ssaN*, a gene of *Salmonella* pathogenicity island 2 (SPI-2) (60), might play a role in the interaction of *Salmonella* with the animal host. The *ssaN* gene product, an ATPase, is essential for the activity of the type III secretion apparatus encoded by SPI-2, which permits survival inside macrophages (61). Formation of SsaN^OFF^ and SsaN^ON^ subpopulations thus raises the possibility that SPI-2 expression inside macrophages may be bistable. Under laboratory conditions, formation of SsaN^OFF^ and SsaN^ON^ lineages is under positive control by OxyR and under negative control by CRP (Figure 3 and Supplementary Figure S3), which may suggest a complex regulation, perhaps responsive to environmental cues. Speculations on the physiological significance of Dam methylation-dependent bistability in housekeeping loci like *holA*, a gene involved in DNA replication, and *dgoR*, a metabolic gene, would be even more premature than for the genes discussed above.

In contrast with our tentative, uncertain speculations on the physiological roles of Dam-dependent bistability in the loci under study, a clear-cut conclusion was obtained in trials aimed at determining whether switching of the loci under study was co-regulated or independent. Indeed, fluorescence microscopy observation of ON and OFF cells in strains that carried pairwise combinations of distinct fluorescent fusions revealed independent switching of seven loci (*ssaN*, *gtr, carA, dgoR, nanA, opvAB* and *holA*) (Figure 4). Dam-dependent control of bistability can thus generate a high degree of transcriptional heterogeneity, as illustrated by the simple calculation that independent switching of *n* bistable loci can produce 2^*n*^ types of cell variants. If this prediction is fulfilled in natural environments, independent switching of the seven loci shown in Figure 4 may be able to produce 128 types of cells. Transcription factors responsive to environmental conditions may further modulate phenotypic heterogeneity by controlling the subpopulation sizes.

Despite its relative success, our survey of the formation of phenotypic cell variants under Dam methylation control may fall short to ponder the contribution of DNA methylation to nonmutational polymorphism. After all, the identification of loci harboring undermethylated GATC sites and the analysis of their expression patterns were done under specific laboratory conditions. Furthermore, the Dam methylase is merely one example among the many DNA methyltransferases present in bacterial genomes (62,63). Undermethylated DNA methyltransferase targets are frequently detected in SMRT sequencing trials (62), thus raising the possibility that DNA methylation hindrance by transcription factors may be a common phenomenon (33). Furthermore, target undermethylation and concomitant lineage formation can be also produced by phase variation in DNA methyltransferase synthesis, a phenomenon that may be widespread among human pathogens (13–15,64). DNA methylation can thus be a powerhouse for the formation of phenotypic variants in bacterial populations, and the known examples of DNA methylation-dependent control of gene expression may be the tip of an iceberg whose magnitude remains unknown.

## Supplementary Material

gkaa730_Supplemental_Files

## References

[1] Finlay B.B., McFadden G. Anti-immunology: evasion of the host immune system by bacterial and viral pathogens. Cell. 2006; 124:767–782. 16497587 10.1016/j.cell.2006.01.034

[2] van Gestel J., Vlamakis H., Kolter R. Division of labor in biofilms: the ecology of cell differentiation. Microbiol. Spectr. 2015; 3:MB-0002–2014. 10.1128/microbiolspec.MB-0002-2014 26104716

[3] Dewachter L., Fauvart M., Michiels J. Bacterial heterogeneity and antibiotic survival: understanding and combatting persistence and heteroresistance. Mol. Cell. 2019; 76:255–267. 31626749 10.1016/j.molcel.2019.09.028

[4] Adam M., Murali B., Glenn N.O., Potter S.S. Epigenetic inheritance based evolution of antibiotic resistance in bacteria. BMC Evol. Biol. 2008; 8:52. 18282299 10.1186/1471-2148-8-52 PMC2262874

[5] Cota I., Sanchez-Romero M.A., Hernandez S.B., Pucciarelli M.G., Garcia-Del Portillo F., Casadesus J. Epigenetic control of Salmonella enterica O-Antigen chain length: A tradeoff between virulence and bacteriophage resistance. PLoS Genet. 2015; 11:e1005667. 26583926 10.1371/journal.pgen.1005667 PMC4652898

[6] Turkington C.J.R., Morozov A., Clokie M.R.J., Bayliss C.D. Phage-resistant phase-variant sub-populations mediate herd immunity against bacteriophage invasion of bacterial meta-populations. Front. Microbiol. 2019; 10:1473. 31333609 10.3389/fmicb.2019.01473 PMC6625227

[7] Kussell E., Leibler S. Phenotypic diversity, population growth, and information in fluctuating environments. Science. 2005; 309:2075–2078. 16123265 10.1126/science.1114383

[8] Wolf D.M., Vazirani V.V., Arkin A.P. A microbial modified prisoner's dilemma game: how frequency-dependent selection can lead to random phase variation. J. Theor. Biol. 2005; 234:255–262. 15757682 10.1016/j.jtbi.2004.11.021

[9] Schroter L., Dersch P. Phenotypic diversification of microbial pathogens – cooperating and preparing for the future. J. Mol. Biol. 2019; 431:4645–4655. 31260693 10.1016/j.jmb.2019.06.024

[10] Veening J.W., Smits W.K., Kuipers O.P. Bistability, epigenetics, and bet-hedging in bacteria. Annu. Rev. Microbiol. 2008; 62:193–210. 18537474 10.1146/annurev.micro.62.081307.163002

[11] Casadesus J., Low D.A. Programmed heterogeneity: epigenetic mechanisms in bacteria. J. Biol. Chem. 2013; 288:13929–13935. 23592777 10.1074/jbc.R113.472274 PMC3656251

[12] Sanchez-Romero M.A., Casadesus J. The bacterial epigenome. Nat. Rev. Microbiol. 2020; 18:7–20. 31728064 10.1038/s41579-019-0286-2

[13] Phillips Z.N., Husna A.U., Jennings M.P., Seib K.L., Atack J.M. Phasevarions of bacterial pathogens - phase-variable epigenetic regulators evolving from restriction-modification systems. Microbiology. 2019; 165:917–928. 30994440 10.1099/mic.0.000805

[14] De Ste Croix M., Vacca I., Kwun M.J., Ralph J.D., Bentley S.D., Haigh R., Croucher N.J., Oggioni M.R. Phase-variable methylation and epigenetic regulation by type I restriction-modification systems. FEMS Microbiol. Rev. 2017; 41:S3–S15. 28830092 10.1093/femsre/fux025

[15] Vasu K., Nagaraja V. Diverse functions of restriction-modification systems in addition to cellular defense. Microbiol. Mol. Biol. Rev. 2013; 77:53–72. 23471617 10.1128/MMBR.00044-12 PMC3591985

[16] Adhikari S., Curtis P.D. DNA methyltransferases and epigenetic regulation in bacteria. FEMS Microbiol. Rev. 2016; 40:575–591. 27476077 10.1093/femsre/fuw023

[17] Marinus M.G. Neidhardt F.C., Curtiss R., Ingraham J.L., Lin E.C.C., Low K.B., Magasanik B., Reznikoff W.S., Riley M., Schaechter M., Umbarger H.E. Methylation of DNA. Escherichia coli and Salmonella: Cellular and Molecular Biology. 1996; Washington, D. C. ASM Press 782–791.

[18] Wion D., Casadesus J. N6-methyl-adenine: an epigenetic signal for DNA-protein interactions. Nat. Rev. Microbiol. 2006; 4:183–192. 16489347 10.1038/nrmicro1350 PMC2755769

[19] Mouammine A., Collier J. The impact of DNA methylation in Alphaproteobacteria. Mol. Microbiol. 2018; 110:1–10. 29995343 10.1111/mmi.14079

[20] Lobner-Olesen A., Skovgaard O., Marinus M.G. Dam methylation: coordinating cellular processes. Curr. Opin. Microbiol. 2005; 8:154–160. 15802246 10.1016/j.mib.2005.02.009

[21] Peterson S.N., Reich N.O. GATC flanking sequences regulate Dam activity: evidence for how Dam specificity may influence pap expression. J. Mol. Biol. 2006; 355:459–472. 16321401 10.1016/j.jmb.2005.11.003

[22] Low D.A., Casadesus J. Clocks and switches: bacterial gene regulation by DNA adenine methylation. Curr. Opin. Microbiol. 2008; 11:106–112. 18396448 10.1016/j.mib.2008.02.012

[23] van der Woude M., Braaten B., Low D. Epigenetic phase variation of the *pap* operon in *Escherichia coli*. Trends Microbiol. 1996; 4:5–9. 8824788 10.1016/0966-842x(96)81498-3

[24] Hernday A., Krabbe M., Braaten B., Low D. Self-perpetuating epigenetic pili switches in bacteria. Proc. Natl. Acad. Sci. U.S.A. 2002; 29:29. 10.1073/pnas.182427199 PMC139910 12202745

[25] Hernday A., Braaten B., Low D. The intricate workings of a bacterial epigenetic switch. Adv. Exp. Med. Biol. 2004; 547:83–89. 15230094 10.1007/978-1-4419-8861-4_7

[26] Haagmans W., van der Woude M. Phase variation of Ag43 in *Escherichia coli*: Dam-dependent methylation abrogates OxyR binding and OxyR-mediated repression of transcription. Mol. Microbiol. 2000; 35:877–887. 10692164 10.1046/j.1365-2958.2000.01762.x

[27] Brunet Y.R., Bernard C.S., Gavioli M., Lloubes R., Cascales E. An epigenetic switch involving overlapping Fur and DNA methylation optimizes expression of a type VI secretion gene cluster. PLoS Genet. 2011; 7:e1002205. 21829382 10.1371/journal.pgen.1002205 PMC3145626

[28] Cota I., Bunk B., Sproer C., Overmann J., Konig C., Casadesus J. OxyR-dependent formation of DNA methylation patterns in OpvAB^OFF^ and OpvAB^ON^ cell lineages of *Salmonella enterica*. Nucleic Acids Res. 2016; 44:3595–3609. 26687718 10.1093/nar/gkv1483 PMC4856963

[29] Broadbent S.E., Davies M.R., van der Woude M.W. Phase variation controls expression of *Salmonella* lipopolysaccharide modification genes by a DNA methylation-dependent mechanism. Mol. Microbiol. 2010; 77:337–353. 20487280 10.1111/j.1365-2958.2010.07203.x PMC2909390

[30] Garcia-Pastor L., Sanchez-Romero M.A., Gutierrez G., Puerta-Fernandez E., Casadesus J. Formation of phenotypic lineages in *Salmonella enterica* by a pleiotropic fimbrial switch. PLoS Genet. 2018; 14:e1007677. 30252837 10.1371/journal.pgen.1007677 PMC6173445

[31] Garcia-Pastor L., Sanchez-Romero M.A., Jakomin M., Puerta-Fernandez E., Casadesus J. Regulation of bistability in the *std* fimbrial operon of *Salmonella enterica* by DNA adenine methylation and transcription factors HdfR, StdE and StdF. Nucleic Acids Res. 2019; 47:7929–7941. 31216025 10.1093/nar/gkz530 PMC6735912

[32] Dalia A.B., Lazinski D.W., Camilli A. Characterization of undermethylated sites in *Vibrio cholerae*. J. Bacteriol. 2013; 195:2389–2399. 23504020 10.1128/JB.02112-12 PMC3650525

[33] Ardissone S., Redder P., Russo G., Frandi A., Fumeaux C., Patrignani A., Schlapbach R., Falquet L., Viollier P.H. Cell cycle constraints and environmental control of local DNA hypomethylation in alpha-Proteobacteria. PLos Genet. 2016; 12:e1006499. 27997543 10.1371/journal.pgen.1006499 PMC5172544

[34] Flusberg B.A., Webster D.R., Lee J.H., Travers K.J., Olivares E.C., Clark T.A., Korlach J., Turner S.W. Direct detection of DNA methylation during single-molecule, real-time sequencing. Nat. Methods. 2010; 7:461–465. 20453866 10.1038/nmeth.1459 PMC2879396

[35] Marinus M.G., Poteete A., Arraj J.A. Correlation of DNA adenine methylase activity with spontaneous mutability in *Escherichia coli* K-12. Gene. 1984; 28:123–125. 6376282 10.1016/0378-1119(84)90095-7

[36] Hautefort I., Proenca M.J., Hinton J.C. Single-copy green fluorescent protein gene fusions allow accurate measurement of *Salmonella* gene expression in vitro and during infection of mammalian cells. Appl. Environ. Microbiol. 2003; 69:7480–7491. 14660401 10.1128/AEM.69.12.7480-7491.2003 PMC310007

[37] Datsenko K.A., Wanner B.L. One-step inactivation of chromosomal genes in *Escherichia coli* K-12 using PCR products. Proc. Natl. Acad. Sci. U.S.A. 2000; 97:6640–6645. 10829079 10.1073/pnas.120163297 PMC18686

[38] Schmieger H. Phage P22-mutants with increased or decreased transduction abilities. Mol. Gen. Genet. 1972; 119:75–88. 4564719 10.1007/BF00270447

[39] Garzon A., Cano D.A., Casadesus J. Role of Erf recombinase in P22-mediated plasmid transduction. Genetics. 1995; 140:427–434. 7498725 10.1093/genetics/140.2.427 PMC1206623

[40] Torreblanca J., Marques S., Casadesus J. Synthesis of FinP RNA by plasmids F and pSLT is regulated by DNA adenine methylation. Genetics. 1999; 152:31–45. 10408954 10.1093/genetics/152.1.31 PMC1460579

[41] Zyskind J.W., Smith D.W. Nucleotide sequence of the *Salmonella typhimurium* origin of DNA replication. Proc. Natl. Acad. Sci. U.S.A. 1980; 77:2460–2464. 6248850 10.1073/pnas.77.5.2460 PMC349419

[42] Hammer Ø., Harper D.A.T., Ryan P.D. PAST: Paleontological statistics software package for education and data analysis. Palaeontol. Electron. 2001; 4:9.

[43] Grant C.E., Bailey T.L., Noble W.S. FIMO: scanning for occurrences of a given motif. Bioinformatics. 2011; 27:1017–1018. 21330290 10.1093/bioinformatics/btr064 PMC3065696

[44] Sanchez-Romero M.A., Casadesus J. Contribution of SPI-1 bistability to *Salmonella enterica* cooperative virulence: insights from single cell analysis. Sci. Rep. 2018; 8:14875. 30291285 10.1038/s41598-018-33137-z PMC6173691

[45] Beuzon C.R., Banks G., Deiwick J., Hensel M., Holden D.W. pH-dependent secretion of SseB, a product of the SPI-2 type III secretion system of *Salmonella typhimurium*. Mol. Microbiol. 1999; 33:806–816. 10447889 10.1046/j.1365-2958.1999.01527.x

[46] Saier M.H. Jr, Ramseier T.M. The catabolite repressor/activator (Cra) protein of enteric bacteria. J. Bacteriol. 1996; 178:3411–3417. 8655535 10.1128/jb.178.12.3411-3417.1996 PMC178107

[47] Brunet Y.R., Bernard C.S., Cascales E. Fur-Dam regulatory interplay at an internal promoter of the enteroaggregative *Escherichia coli* Type VI Secretion *sci1* Gene Cluster. J. Bacteriol. 2020; 202:e00075-20. 32152218 10.1128/JB.00075-20 PMC7186456

[48] Cota I., Blanc-Potard A.B., Casadesus J. *STM2209-STM2208 (opvAB)*: a phase variation locus of *Salmonella enterica* involved in control of O-antigen chain length. PLoS One. 2012; 7:e36863. 22606300 10.1371/journal.pone.0036863 PMC3350482

[49] Bailey T.L., Boden M., Buske F.A., Frith M., Grant C.E., Clementi L., Ren J., Li W.W., Noble W.S. MEME SUITE: tools for motif discovery and searching. Nucleic Acids Res. 2009; 37:W202–W208. 19458158 10.1093/nar/gkp335 PMC2703892

[50] Casadesus J., Low D. Epigenetic gene regulation in the bacterial world. Microbiol. Mol. Biol. Rev. 2006; 70:830–856. 16959970 10.1128/MMBR.00016-06 PMC1594586

[51] Silva-Rocha R., de Lorenzo V. Noise and robustness in prokaryotic regulatory networks. Annu. Rev. Microbiol. 2010; 64:257–275. 20825349 10.1146/annurev.micro.091208.073229

[52] Raser J.M., O'Shea E.K. Noise in gene expression: origins, consequences, and control. Science. 2005; 309:2010–2013. 16179466 10.1126/science.1105891 PMC1360161

[53] Dubnau D., Losick R. Bistability in bacteria. Mol. Microbiol. 2006; 61:564–572. 16879639 10.1111/j.1365-2958.2006.05249.x

[54] Graumann P.L. Different genetic programmes within identical bacteria under identical conditions: the phenomenon of bistability greatly modifies our view on bacterial populations. Mol. Microbiol. 2006; 61:560–563. 16879638 10.1111/j.1365-2958.2006.05264.x

[55] Garcia-Pastor L., Puerta-Fernandez E., Casadesus J. Bistability and phase variation in *Salmonella enterica*. Biochim. Biophys. Acta Gene Regul. Mech. 2019; 1862:752–758. 29369799 10.1016/j.bbagrm.2018.01.003

[56] Torreblanca J., Casadesus J. DNA adenine methylase mutants of *Salmonella typhimurium* and a novel dam- regulated locus. Genetics. 1996; 144:15–26. 8878670 10.1093/genetics/144.1.15 PMC1207489

[57] Balbontin R., Rowley G., Pucciarelli M.G., Lopez-Garrido J., Wormstone Y., Lucchini S., Garcia-Del Portillo F., Hinton J.C., Casadesus J. DNA adenine methylation regulates virulence gene expression in *Salmonella enterica* serovar Typhimurium. J. Bacteriol. 2006; 188:8160–8168. 16997949 10.1128/JB.00847-06 PMC1698197

[58] Ringquist S., Smith C.L. The *Escherichia coli* chromosome contains specific, unmethylated *dam* and *dcm* sites. Proc. Natl. Acad. Sci. U.S.A. 1992; 89:4539–4543. 1584789 10.1073/pnas.89.10.4539 PMC49118

[59] Hale W.B., van der Woude M.W., Low D.A. Analysis of nonmethylated GATC sites in the *Escherichia coli* chromosome and identification of sites that are differentially methylated in response to environmental stimuli. J. Bacteriol. 1994; 176:3438–3441. 8195106 10.1128/jb.176.11.3438-3441.1994 PMC205523

[60] Jennings E., Thurston T.L.M., Holden D.W. *Salmonella* SPI-2 type III secretion system effectors: Molecular mechanisms and physiological consequences. Cell Host Microbe. 2017; 22:217–231. 28799907 10.1016/j.chom.2017.07.009

[61] Yoshida Y., Miki T., Ono S., Haneda T., Ito M., Okada N. Functional characterization of the type III secretion ATPase SsaN encoded by *Salmonella* pathogenicity island 2. PLoS One. 2014; 9:e94347. 24722491 10.1371/journal.pone.0094347 PMC3983159

[62] Blow M.J., Clark T.A., Daum C.G., Deutschbauer A.M., Fomenkov A., Fries R., Froula J., Kang D.D., Malmstrom R.R., Morgan R.D. et al. The epigenomic landscape of prokaryotes. PLoS Genet. 2016; 12:e1005854. 26870957 10.1371/journal.pgen.1005854 PMC4752239

[63] Roberts R.J., Vincze T., Posfai J., Macelis D. REBASE – a database for DNA restriction and modification: enzymes, genes and genomes. Nucleic Acids Res. 2015; 43:D298–299. 25378308 10.1093/nar/gku1046 PMC4383893

[64] Srikhanta Y.N., Fox K.L., Jennings M.P. The phasevarion: phase variation of type III DNA methyltransferases controls coordinated switching in multiple genes. Nat. Rev. Microbiol. 2009; 8:196–206. 10.1038/nrmicro2283 20140025

