# Supplementary material for: Contribution of DNA adenine methylation to gene expression heterogeneity in Salmonella enterica
Source: Nucleic Acids Res. 2020 Sep 21;48(21):11857–67. doi: 10.1093/nar/gkaa730 (PMC7708049; doi:10.1093/nar/gkaa730)

Contribution of DNA adenine methylation to gene expression heterogeneity in *Salmonella enterica*
M. A. Sánchez-Romero, D. R. Olivenza, G. Gutiérrez and J. Casadesús

Figure S1. Loci discarded from further analysis due to low or absent expression of GFP fusions. Cultures were grown at 37ºC in LB under aerobiosis, LB under microaerophilia and intracellular salts medium (ISM). Dot plots represent the GFP fluorescence intensity versus the forward scatter cell or the cell size in arbitrary units.

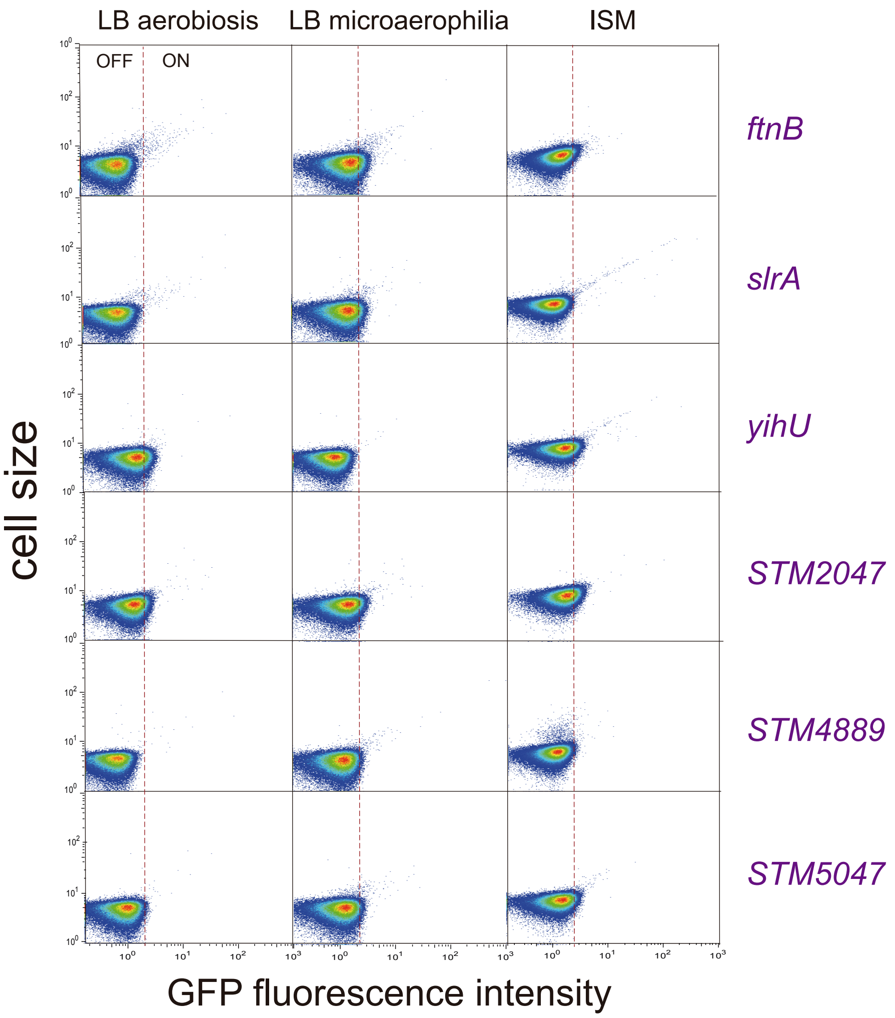

Supplement: gkaa730_Supplemental_Files [file gkaa730_supplemental_files.zip › Figure S1.pdf]
