# Supplementary material for: Contribution of DNA adenine methylation to gene expression heterogeneity in Salmonella enterica
Source: Nucleic Acids Res. 2020 Sep 21;48(21):11857–67. doi: 10.1093/nar/gkaa730 (PMC7708049; doi:10.1093/nar/gkaa730)
Supplement: gkaa730_Supplemental_Files [file gkaa730_supplemental_files.zip › Figure S2.pdf]

Contribution of DNA adenine methylation to gene expression heterogeneity in *Salmonella enterica*
M. A. Sánchez-Romero, D. R. Olivenza, G. Gutiérrez and J. Casadesús

**Figure S2.** Single cell analysis of Dam methylation-dependent regulation of gene expression. Flow cytometry analysis was performed in the wild type (left column and red line in the histograms), in a *dam* mutant (middle column and blue line in the histograms) and in a Dam methylase overproducing strain (right column and green line in histograms). Cultures were grown at 37ºC in LB under microaerophilia, LB under aerobiosis and intracellular salts medium (ISM).

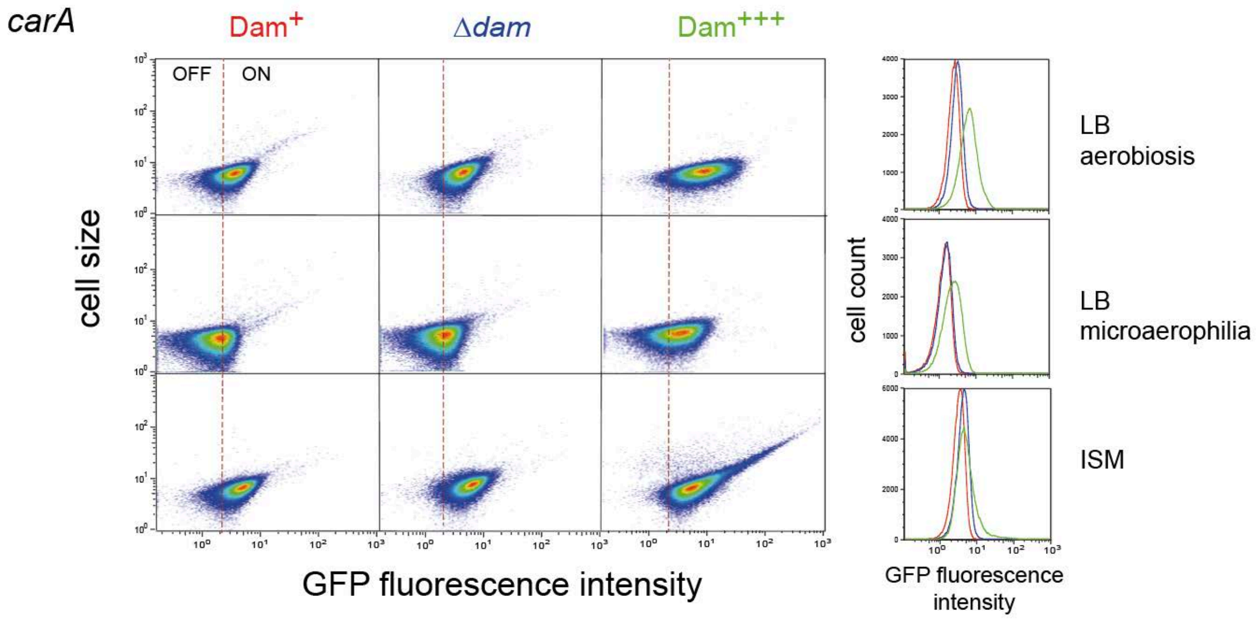


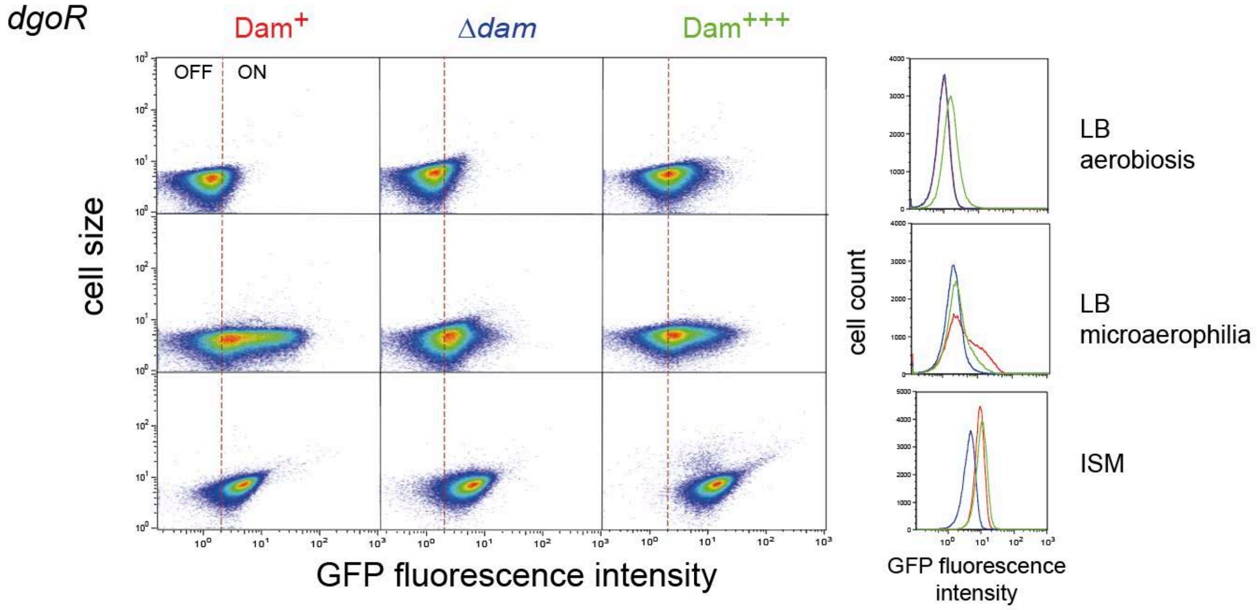

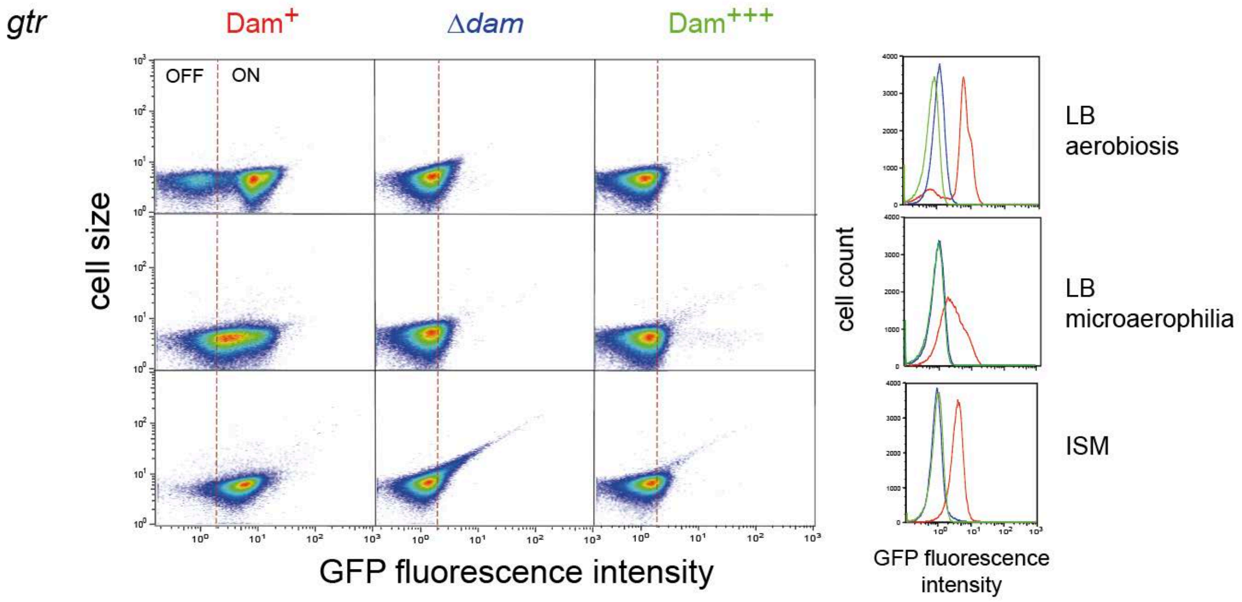
gtr
Dam+
Δdam
Dam+++
OFF
ON
cell size
GFP fluorescence intensity
cell count
GFP fluorescence intensity
LB aerobiosis
LB microaerophilia
ISM

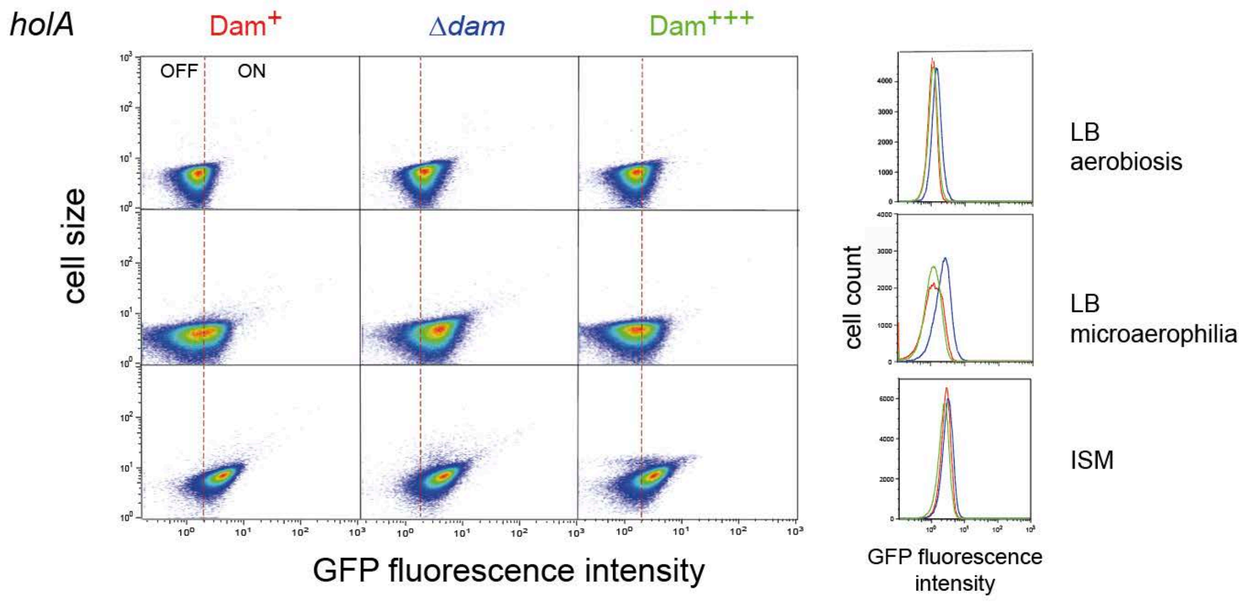
holA
Dam+
Δdam
Dam+++
OFF
ON
cell size
GFP fluorescence intensity
cell count
GFP fluorescence intensity
LB aerobiosis
LB microaerophilia
ISM

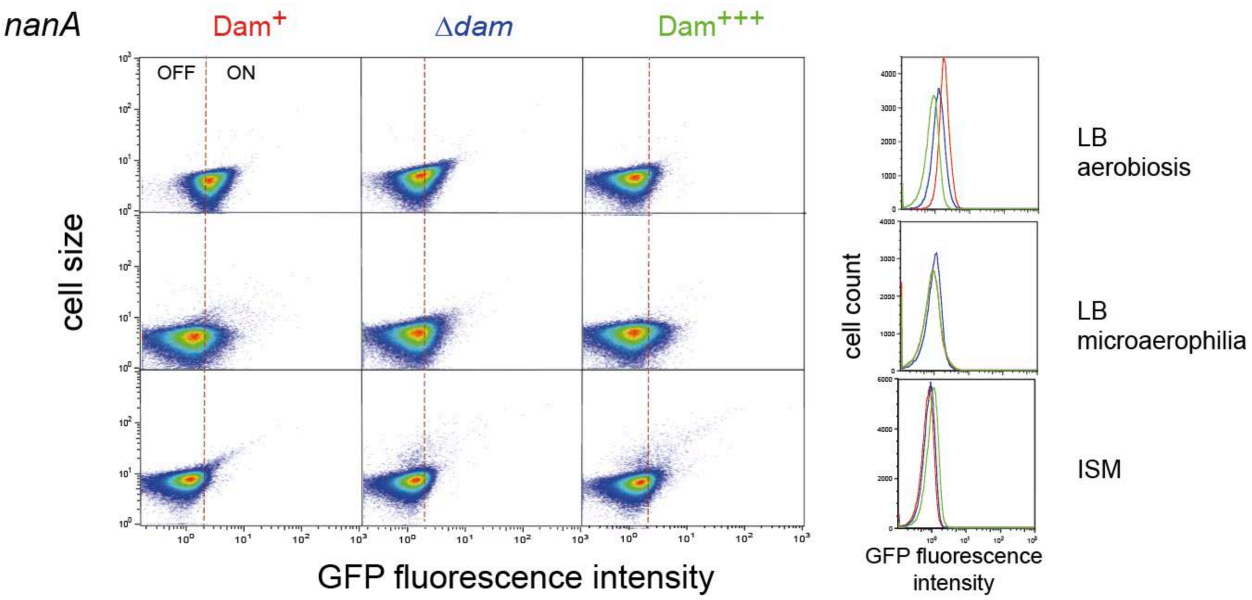
nanA
Dam+
Δdam
Dam+++
OFF
ON
cell size
cell count
LB
aerobiosis
LB
microaerophilia
ISM
GFP fluorescence intensity
GFP fluorescence
intensity

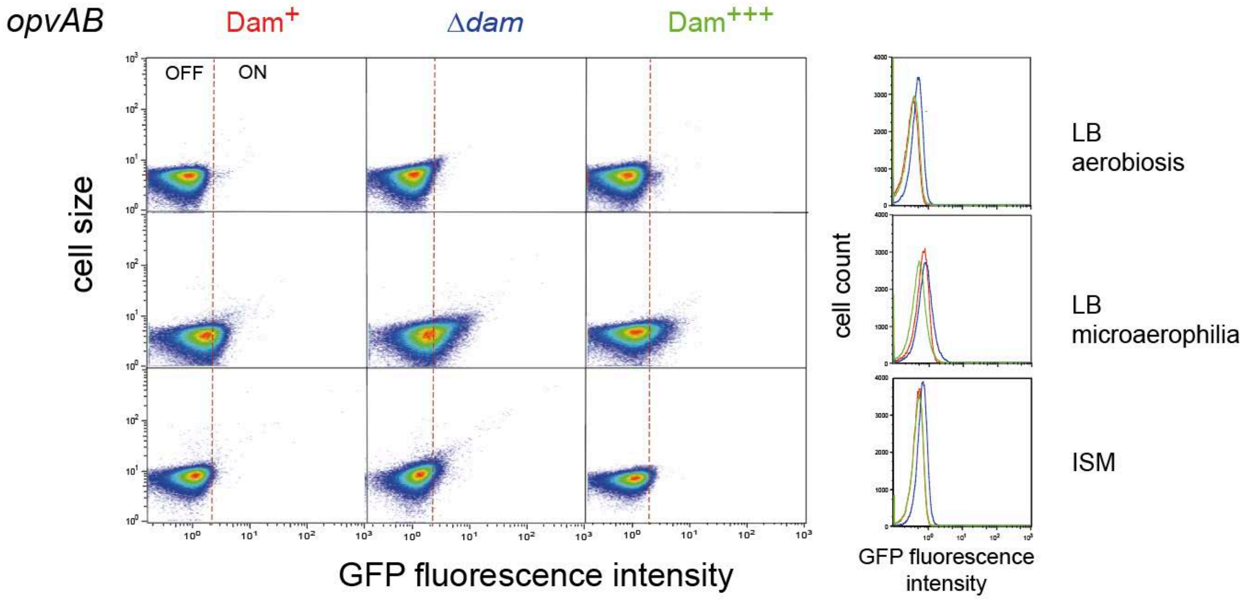
opvAB
Dam+
Δdam
Dam+++
OFF
ON
cell size
cell count
LB
aerobiosis
LB
microaerophilia
ISM
GFP fluorescence intensity
GFP fluorescence
intensity

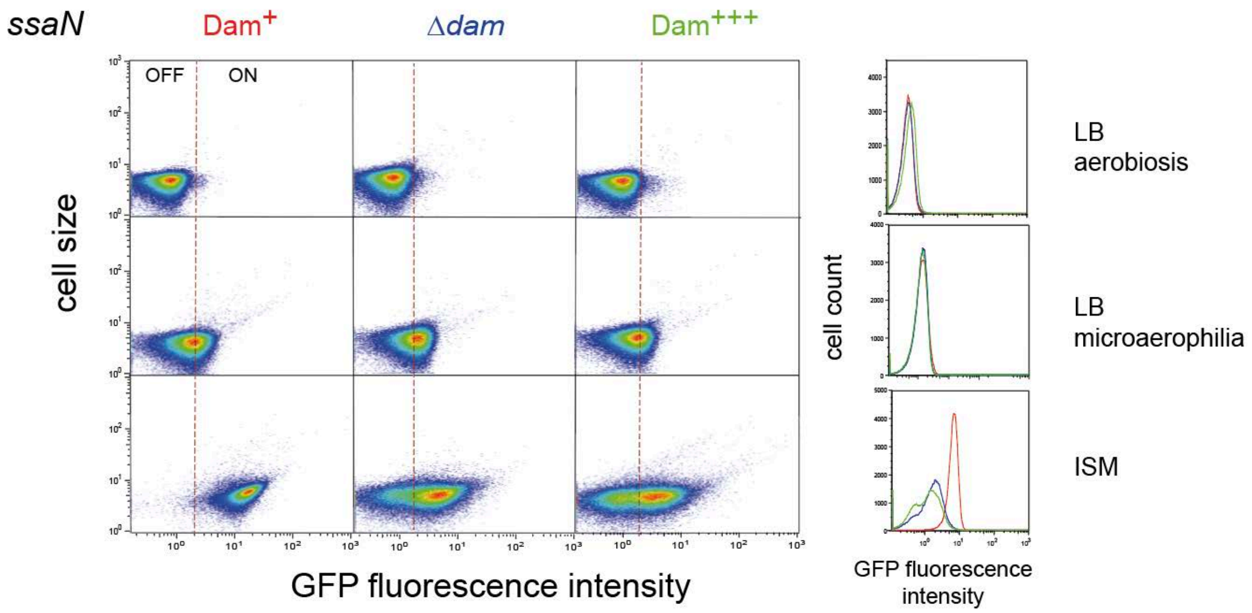
ssaN
Dam+
Δdam
Dam+++
OFF
ON
cell size
GFP fluorescence intensity
cell count
GFP fluorescence intensity
LB aerobiosis
LB microaerophilia
ISM

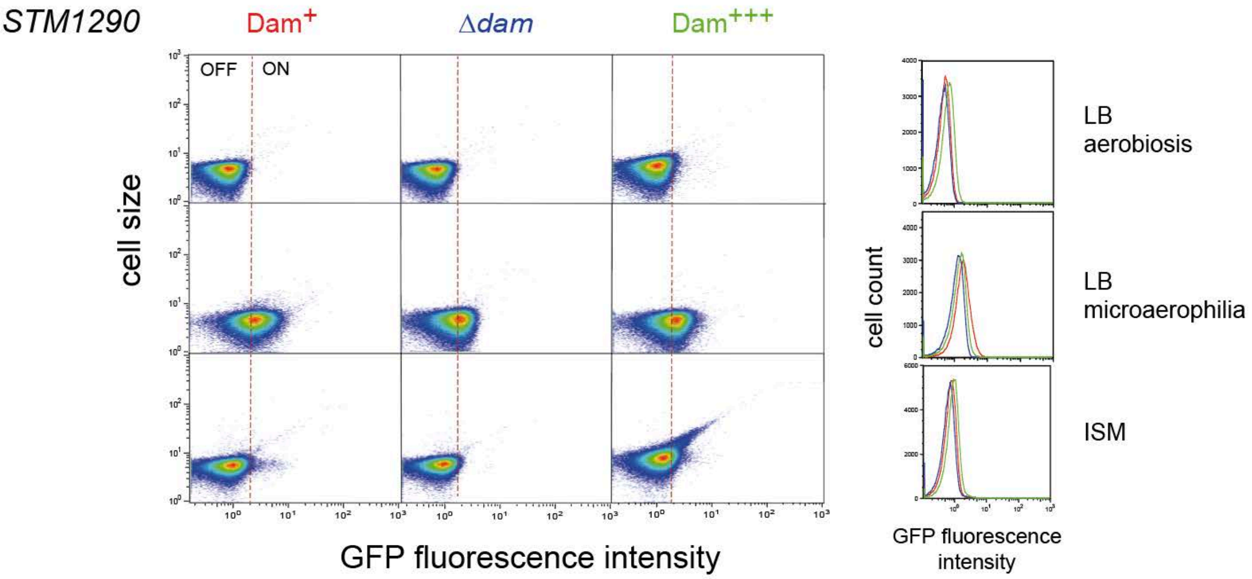
STM1290
Dam+
Δdam
Dam+++
OFF
ON
cell size
GFP fluorescence intensity
cell count
GFP fluorescence intensity
LB aerobiosis
LB microaerophilia
ISM

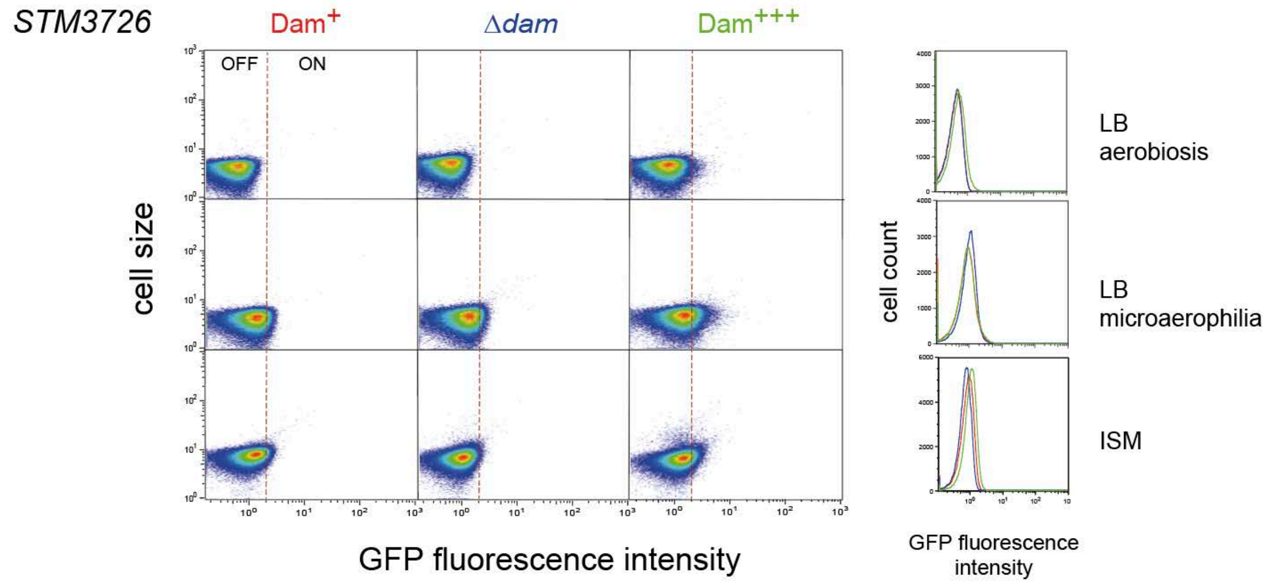
STM3726
Dam+
Δdam
Dam+++
OFF
ON
cell size
GFP fluorescence intensity
cell count
GFP fluorescence intensity
LB aerobiosis
LB microaerophilia
ISM

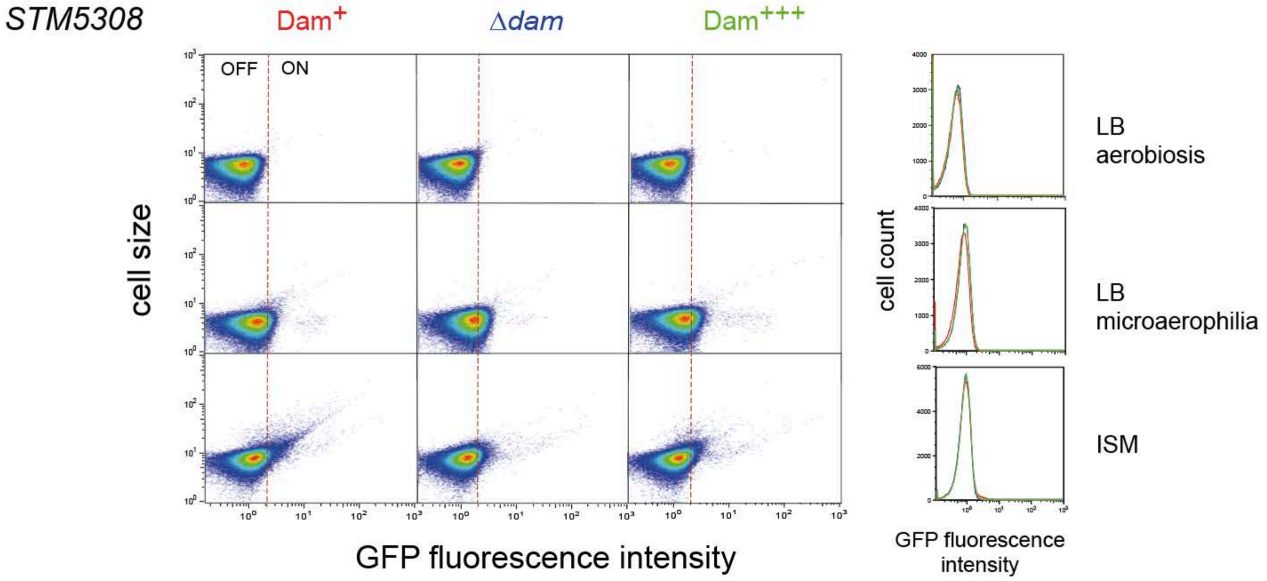
STM5308
Dam+
Δdam
Dam+++
OFF
ON
cell size
GFP fluorescence intensity
cell count
GFP fluorescence intensity
LB aerobiosis
LB microaerophilia
ISM
