# Supplementary material for: Contribution of DNA adenine methylation to gene expression heterogeneity in Salmonella enterica
Source: Nucleic Acids Res. 2020 Sep 21;48(21):11857–67. doi: 10.1093/nar/gkaa730 (PMC7708049; doi:10.1093/nar/gkaa730)
Supplement: gkaa730_Supplemental_Files [file gkaa730_supplemental_files.zip › Figure S3.pdf]

Figure S3. Single cell analysis of gene expression in strains lacking individual transcription factors. GFP fluorescence intensity distribution was measured in cultures grown at 37ºC in LB under aerobiosis, LB under microaerophilia and intracellular salts medium (ISM).

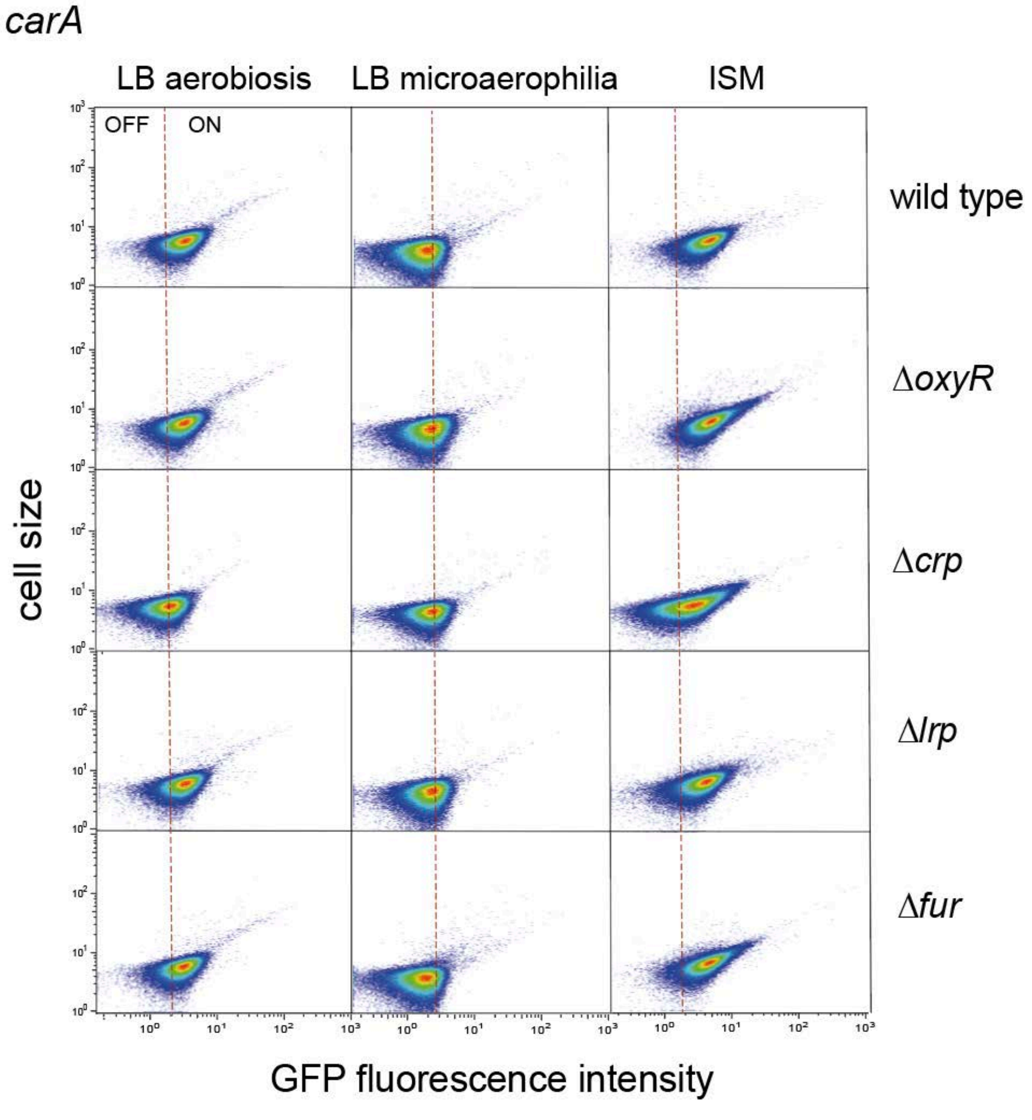

dgoR

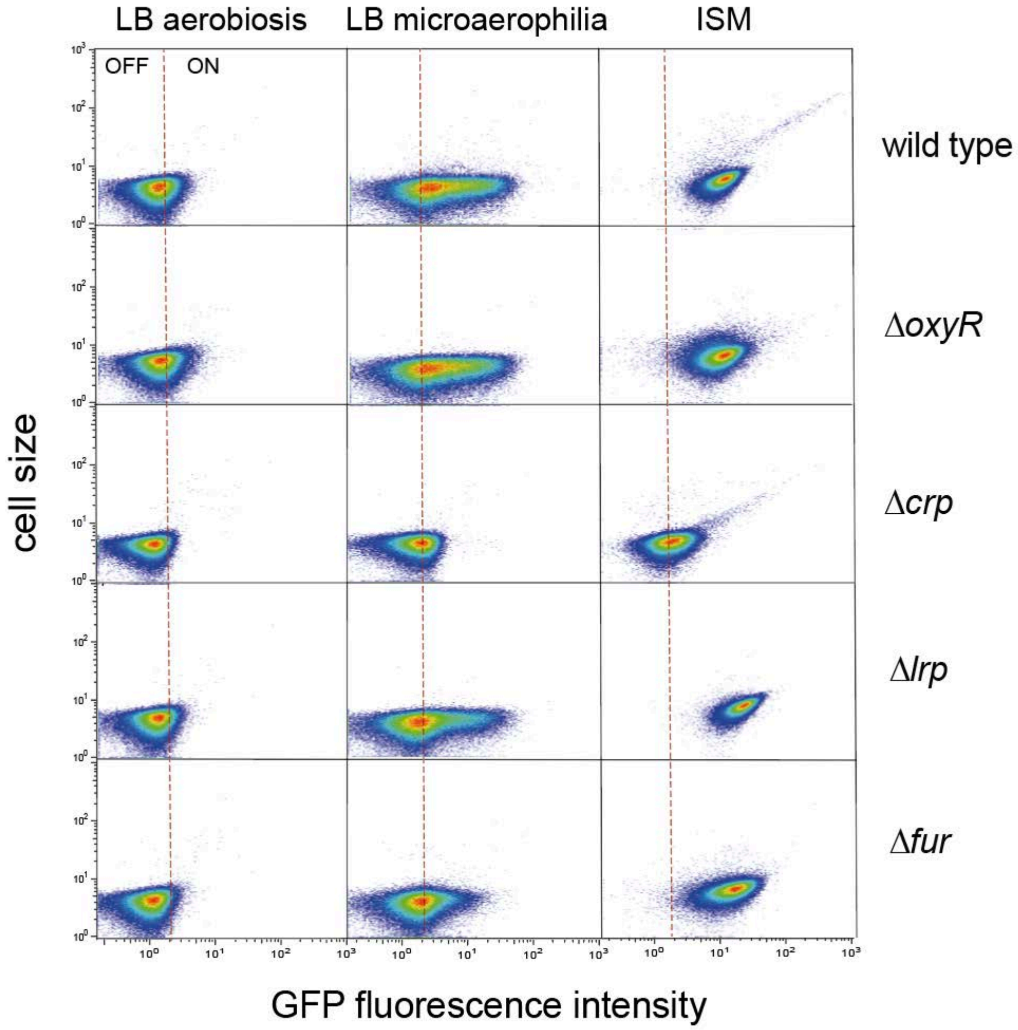

LB aerobiosis
LB microaerophilia
ISM
OFF
ON
wild type
ΔoxyR
Δcrp
Δlrp
Δfur
cell size
GFP fluorescence intensity

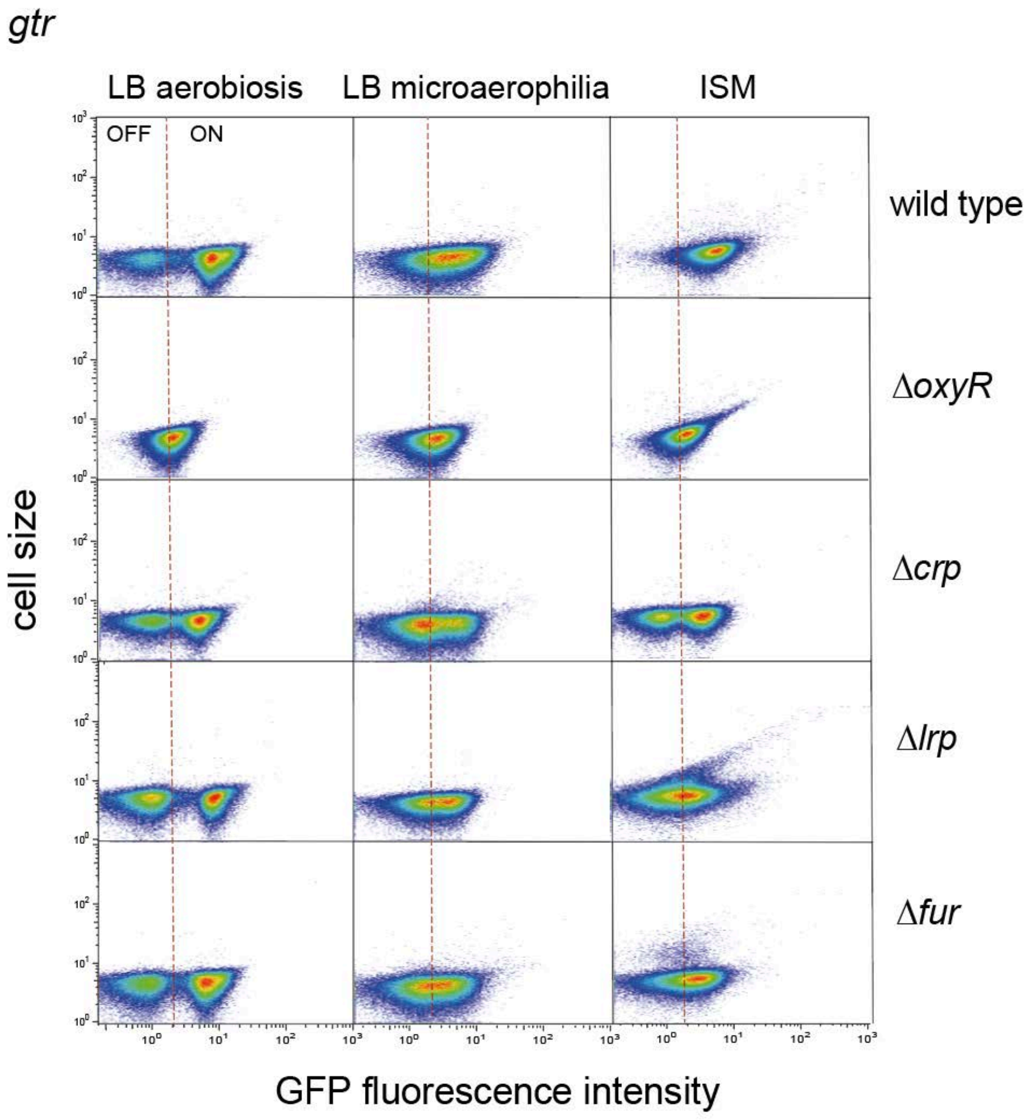
gtr
LB aerobiosis
LB microaerophilia
ISM
OFF
ON
wild type
ΔoxyR
Δcrp
Δlrp
Δfur
cell size
GFP fluorescence intensity

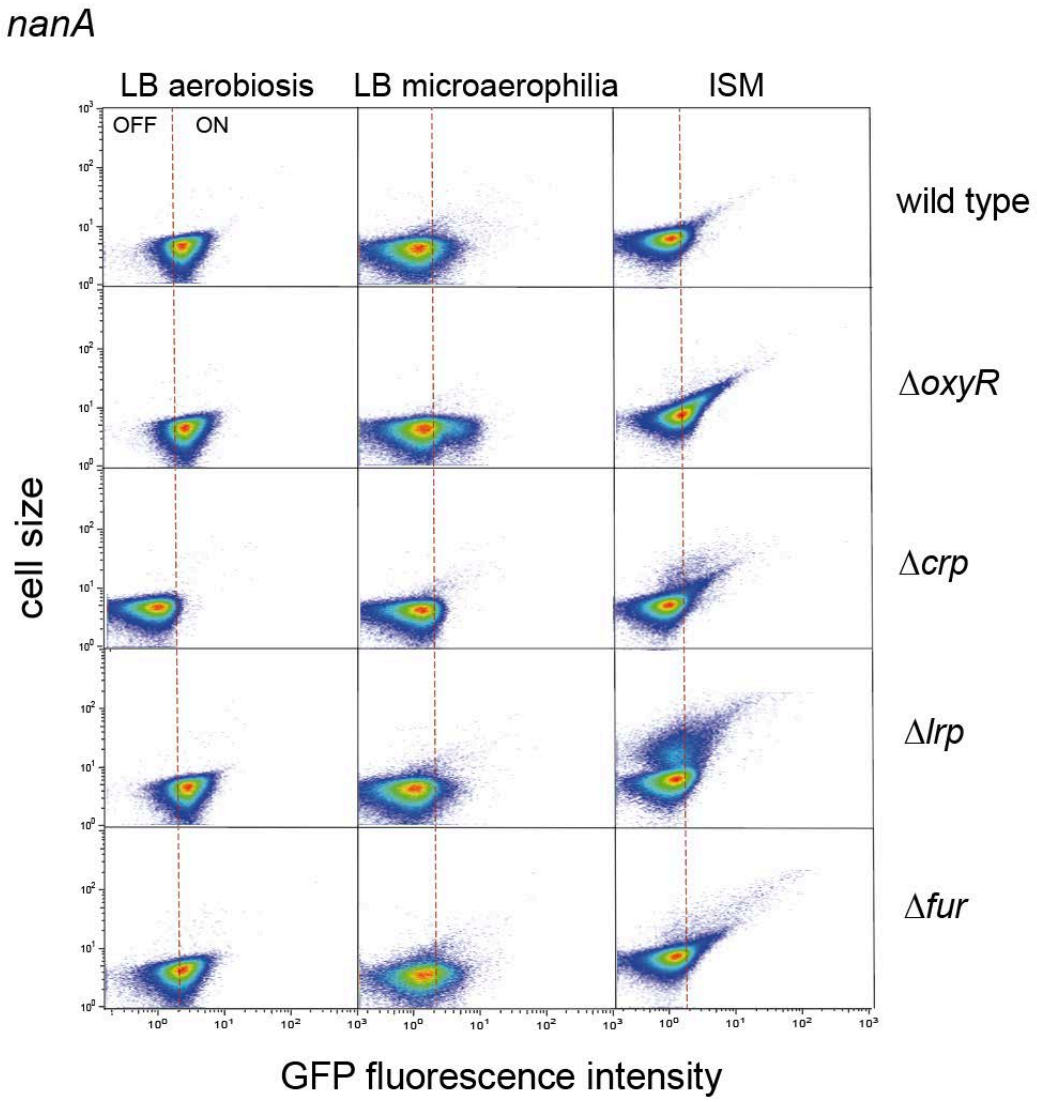

nanA
LB aerobiosis
LB microaerophilia
ISM
OFF
ON
wild type
ΔoxyR
Δcrp
Δlrp
Δfur
cell size
GFP fluorescence intensity

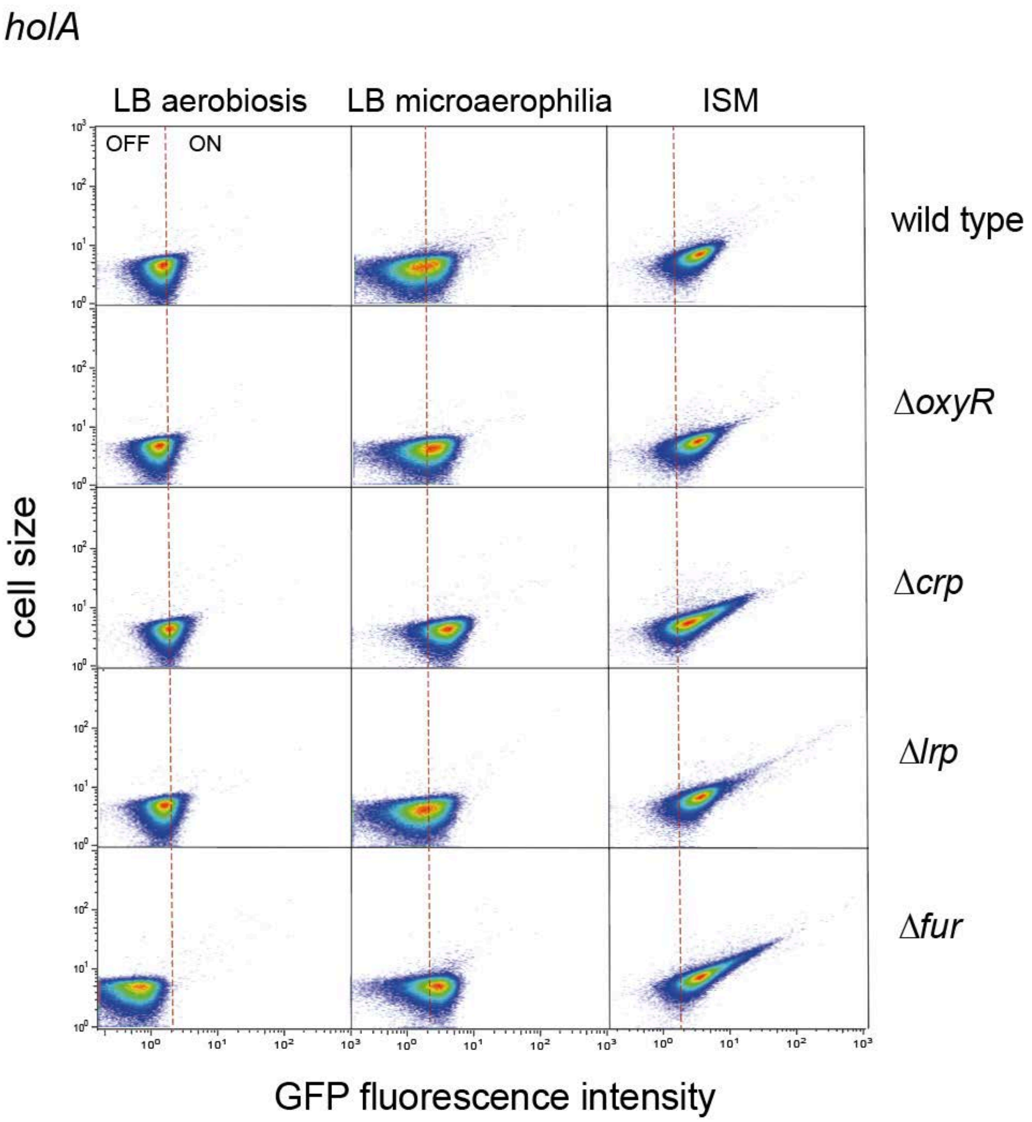
holA
LB aerobiosis
LB microaerophilia
ISM
OFF
ON
wild type
ΔoxyR
Δcrp
Δlrp
Δfur
cell size
GFP fluorescence intensity

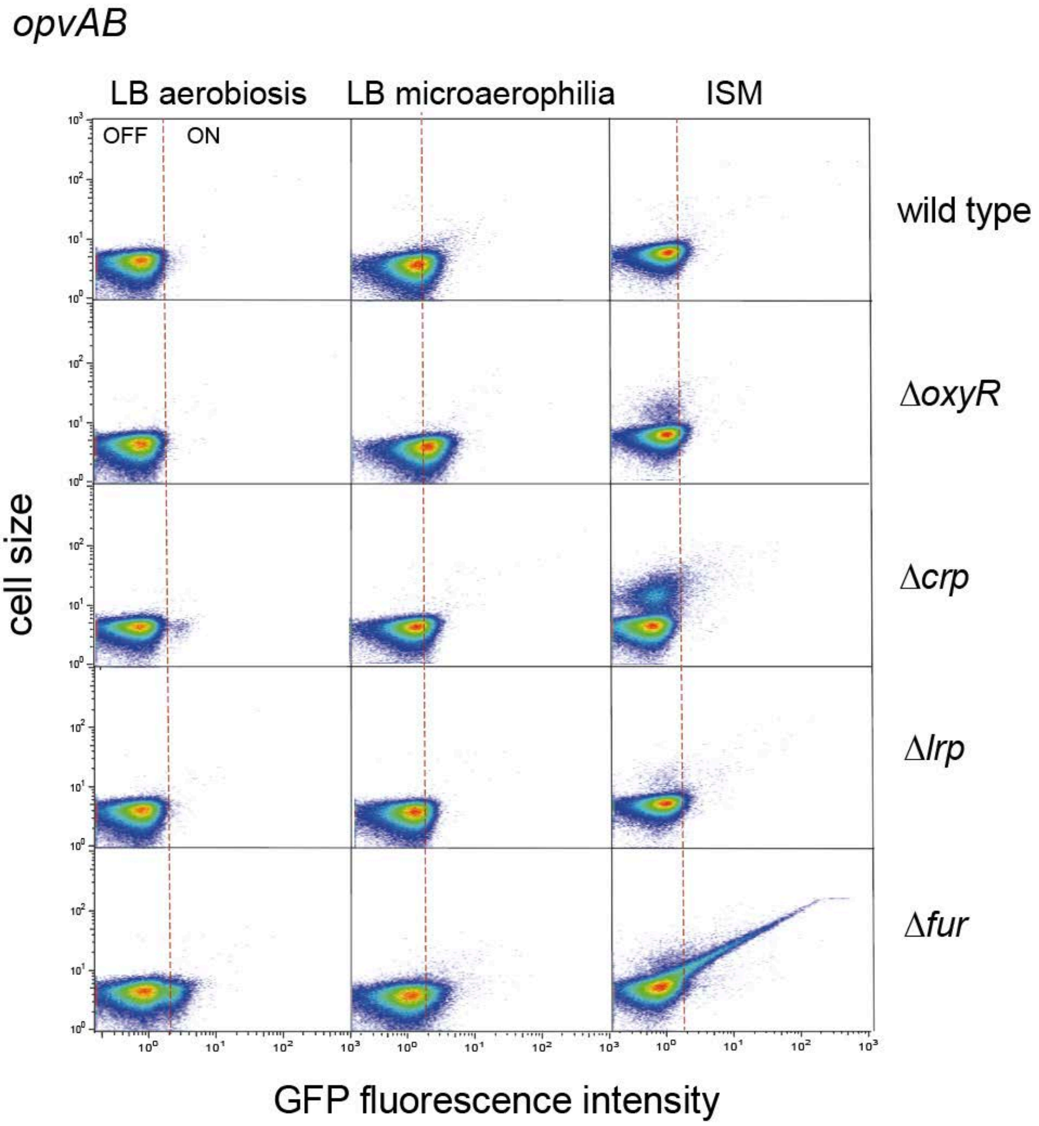
opvAB
LB aerobiosis
LB microaerophilia
ISM
OFF
ON
wild type
ΔoxyR
Δcrp
Δlrp
Δfur
cell size
GFP fluorescence intensity

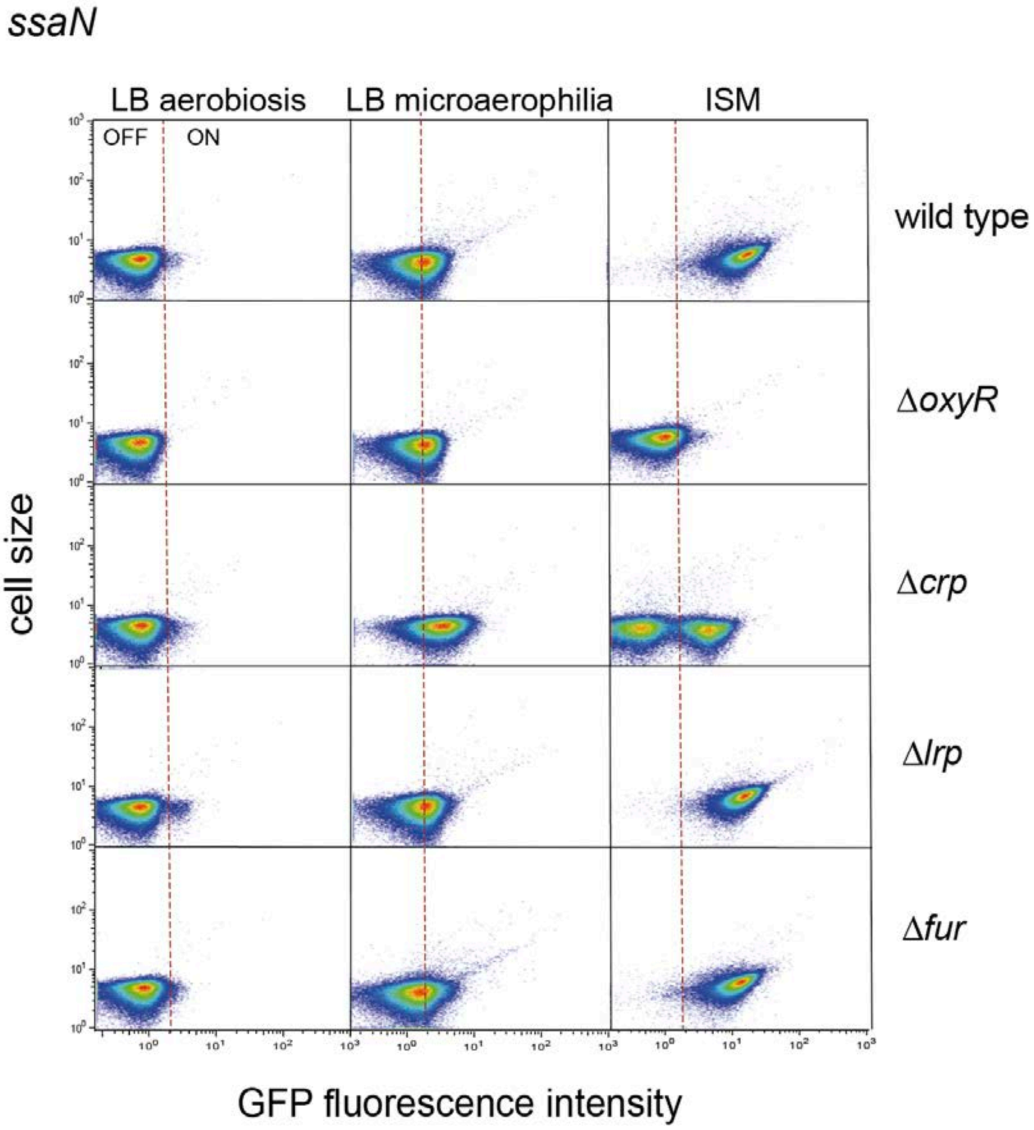

ssaN
LB aerobiosis
LB microaerophilia
ISM
OFF
ON
wild type
ΔoxyR
Δcrp
Δlrp
Δfur
cell size
GFP fluorescence intensity

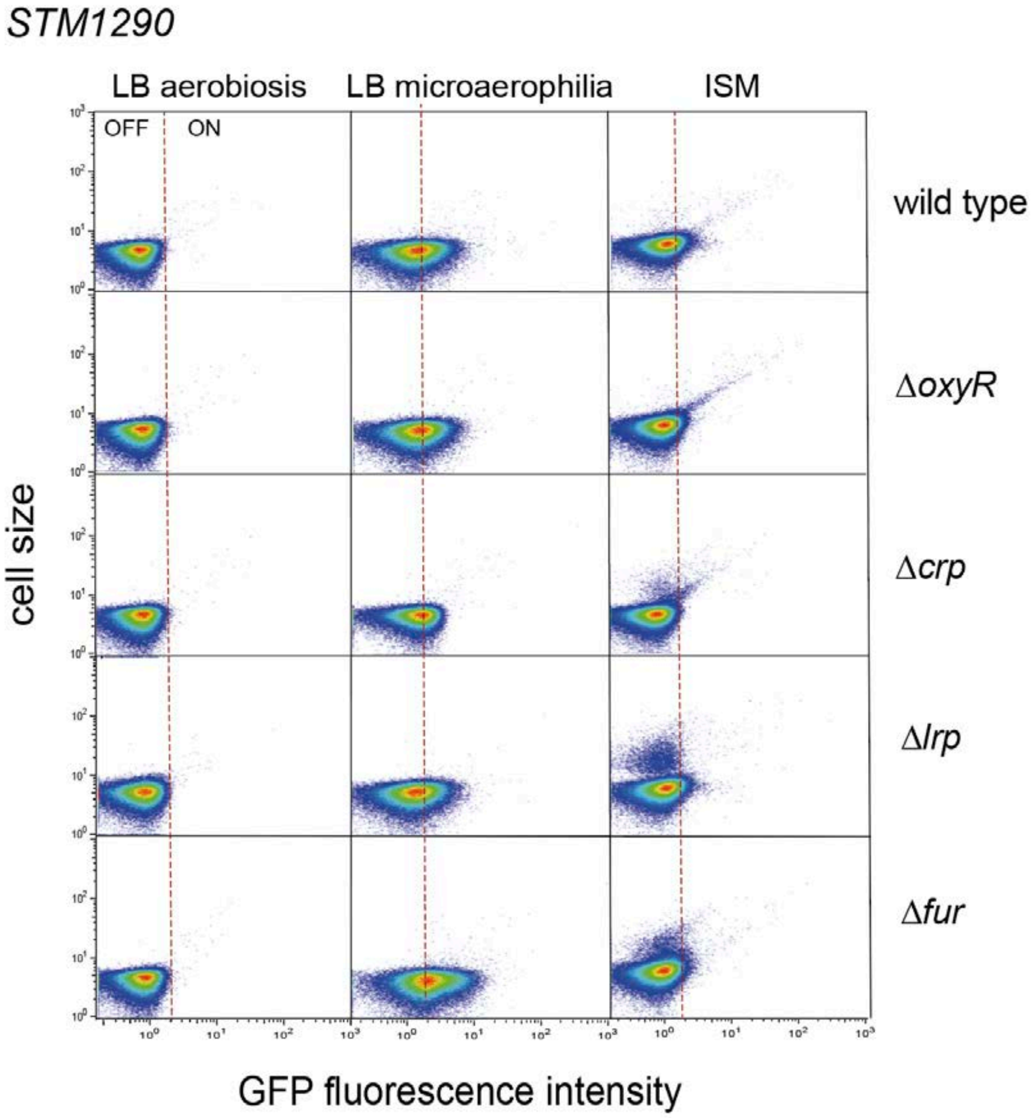
STM1290
LB aerobiosis
LB microaerophilia
ISM
OFF
ON
wild type
ΔoxyR
Δcrp
Δlrp
Δfur
cell size
GFP fluorescence intensity

STM3726

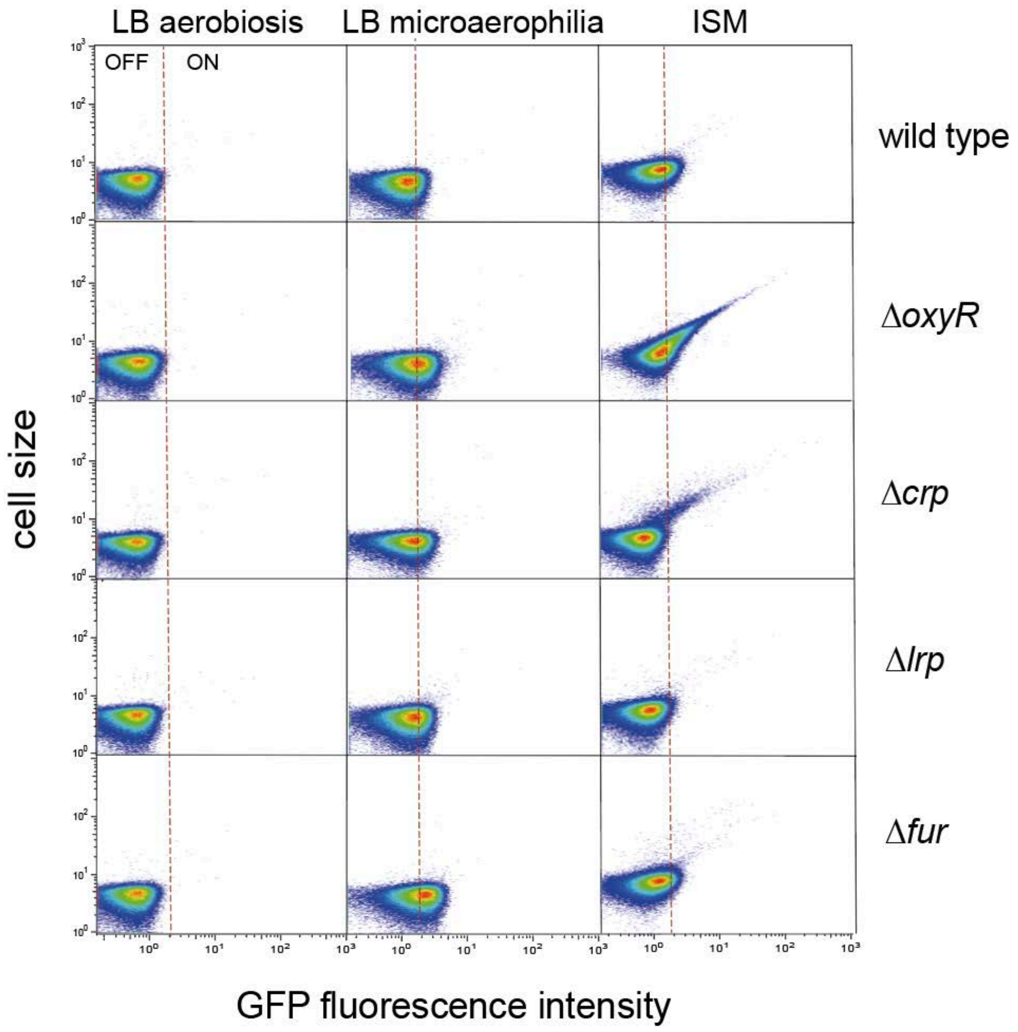

LB aerobiosis
LB microaerophilia
ISM
OFF
ON
wild type
ΔoxyR
Δcrp
Δlrp
Δfur
cell size
GFP fluorescence intensity
