# Supplementary material for: Contribution of DNA adenine methylation to gene expression heterogeneity in Salmonella enterica
Source: Nucleic Acids Res. 2020 Sep 21;48(21):11857–67. doi: 10.1093/nar/gkaa730 (PMC7708049; doi:10.1093/nar/gkaa730)
Supplement: gkaa730_Supplemental_Files [file gkaa730_supplemental_files.zip › Figure S4.pdf]

Contribution of DNA adenine methylation to gene expression heterogeneity in *Salmonella enterica*

M. A. Sánchez-Romero, D. R. Olivenza, G. Gutiérrez and J. Casadesús

Figure S4. Methylation state of the *nanA* promoter and UAS in Crp^{+} (blue) and Crp^{-} (red) backgrounds, inferred from quantitative PCR analysis after digestion with enzymes that cut GATC sequences depending on their methylation state (*Mbo*I and *Dpn*I).

Control

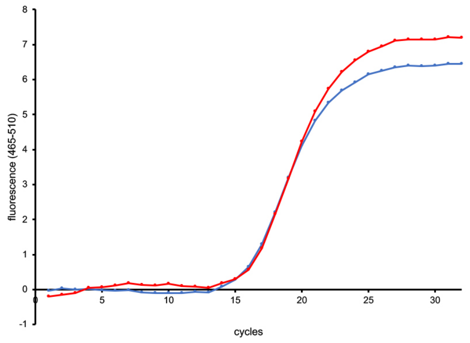

*Mbo*I

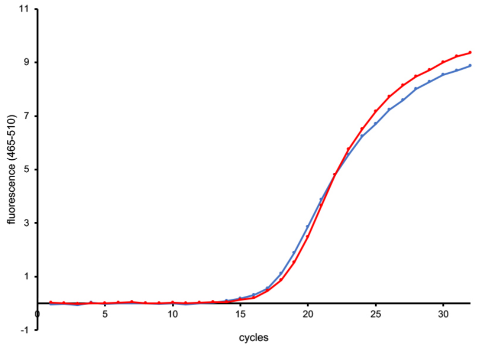

*Dpn*I

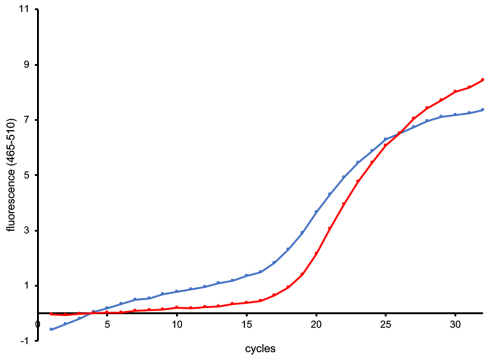

Genomic DNA was isolated by phenol extraction and ethanol precipitation. DNA samples were digested with the endonucleases *Mbo*I and *Dpn*I (New England Biolabs). After digestion, RT-PCR was performed using the DNA samples as templates. The oligonucleotides used were nanA-GATC-F and nanA-GATC-R, described in Table S2.
