# Supplementary material for: Contribution of DNA adenine methylation to gene expression heterogeneity in Salmonella enterica
Source: Nucleic Acids Res. 2020 Sep 21;48(21):11857–67. doi: 10.1093/nar/gkaa730 (PMC7708049; doi:10.1093/nar/gkaa730)
Supplement: gkaa730_Supplemental_Files [file gkaa730_supplemental_files.zip › Table S1.pdf]

M. A. Sánchez-Romero, D. R. Olivenza, G. Gutiérrez and J. Casadesús

Table S1. Strains used in this study

Name	Genotype
SV7155	Δ*dam-231*::Km^R
SV5944	Δ*oxyR*:: Km^R
SV8474	Δ*crp*:: Km^R
SV8332	Δ*lrp*::Km^R
SV9804	*dgoR::gfp*
SV9806	*dgoR::gfp* Δ*dam*
SV9805	*dgoR::gfp* / pTP166
SV9808	*dgoR::gfp* Δ*crp*
SV9809	*dgoR::gfp* Δ*lrp*
SV9807	*dgoR::gfp* Δ*oxyR*
SV9811	*dgoR::gfp* Δ*fur*
SV9892	*carA::gfp*
SV9894	*carA::gfp* Δ*dam*
SV9893	*carA::gfp* / pTP166
SV9896	*carA::gfp* Δ*crp*
SV9897	*carA::gfp* Δ*lrp*
SV9895	*carA::gfp* Δ*oxyR*
SV9899	*carA::gfp* Δ*fur*
SV9876	*ssaN::gfp*
SV9878	*ssaN::gfp* Δ*dam*
SV9877	*ssaN::gfp* / pTP166
SV9880	*ssaN::gfp* Δ*crp*
SV9881	*ssaN::gfp* Δ*lrp*
SV9879	*ssaN::gfp* Δ*oxyR*
SV9883	*ssaN::gfp* Δ*fur*
SV9844	*STM1290::gfp*
SV9846	*STM1290::gfp* Δ*dam*
SV9845	*STM1290::gfp* / pTP166

SV9848	*STM1290::gfp* Δ*crp*
SV9849	*STM1290::gfp* Δ*lrp*
SV9847	*STM1290::gfp* Δ*oxyR*
SV9851	*STM1290::gfp* Δ*fur*
SV9924	*STM5308::gfp*
SV9926	*STM5308::gfp* Δ*dam*
SV9925	*STM5308::gfp* / pTP166
SV9928	*STM5308::gfp* Δ*crp*
SV9929	*STM5308::gfp* Δ*lrp*
SV9927	*STM5308::gfp* Δ*oxyR*
SV9931	*STM5308::gfp* Δ*fur*
SV6727	*opvAB::gfp*
SV9942	*opvAB::gfp* Δ*dam*
SV9941	*opvAB::gfp* / pTP166
SV9944	*opvAB::gfp* Δ*crp*
SV9945	*opvAB::gfp* Δ*lrp*
SV9943	*opvAB::gfp* Δ*oxyR*
SV9947	*opvAB::gfp* Δ*fur*
SV9948	*gtrA::gfp*
SV9950	*gtrA::gfp* Δ*dam*
SV9949	*gtrA::gfp* / pTP166
SV9952	*gtrA::gfp* Δ*crp*
SV9953	*gtrA::gfp* Δ*lrp*
SV9951	*gtrA::gfp* Δ*oxyR*
SV9955	*gtrA::gfp* Δ*fur*
SV9828	*holA::gfp*
SV9830	*holA::gfp* Δ*dam*
SV9829	*holA::gfp* / pTP166
SV9832	*holA::gfp* Δ*crp*
SV9833	*holA::gfp* Δ*lrp*
SV9831	*holA::gfp* Δ*oxyR*
SV9835	*holA::gfp* Δ*fur*
SV9868	*STM3726::gfp*

SV9870	*STM3726::gfp Δdam*
SV9869	*STM3726::gfp* / pTP166
SV9872	*STM3726::gfp Δcrp*
SV9873	*STM3726::gfp Δlrp*
SV9871	*STM3726::gfp ΔoxyR*
SV9875	*STM3726::gfp Δfur*
SV9812	*nanA::gfp*
SV9814	*nanA::gfp Δdam*
SV9813	*nanA::gfp* / pTP166
SV9816	*nanA::gfp Δcrp*
SV9817	*nanA::gfp Δcrp*
SV9819	*nanA::gfp Δlrp*
SV9815	*nanA::gfp ΔoxyR*
SV9819	*nanA::gfp Δfur*
SV9836	*slrA::gfp*
SV9860	*yihU::gfp*
SV9852	*ftnB::gfp*
SV9908	*STM4889::gfp*
SV9820	*STM2047::gfp*
SV10077	Δ*fur*:: Km^{R}
SV10078	*ssaN::gfp gtrA::mCherry*
SV10079	*ssaN::gfp carA::mOrange2*
SV10080	*nanA::gfp ssaN::mCherry*
SV10081	*nanA::gfp ssaN::mOrange2*
SV10082	*opvAB::gfp gtrA::mCherry*
SV10083	*opvAB::gfp ssaN::mOrange2*
SV10084	*holA::gfp gtrA::mCherry*
SV10085	*holA::gfp ssaN::mOrange2*
SV10086	*STM5047::gfp*
SV10087	*dgoR::gfp ssaN::mOrange2*
SV10088	*dgoR::gfp gtrA::mcherry*
