# Supplementary material for: Contribution of DNA adenine methylation to gene expression heterogeneity in Salmonella enterica
Source: Nucleic Acids Res. 2020 Sep 21;48(21):11857–67. doi: 10.1093/nar/gkaa730 (PMC7708049; doi:10.1093/nar/gkaa730)
Supplement: gkaa730_Supplemental_Files [file gkaa730_supplemental_files.zip › Table S2.pdf]

Contribution of DNA adenine methylation to gene expression heterogeneity in *Salmonella enterica*
M. A. Sánchez-Romero, D. R. Olivenza, G. Gutiérrez and J. Casadesús

Table S2. Oligonucleotides used in this study

Name	Sequence (5'-->3')
dgoR-GFP-F	GATCGCCAGCTCGACACGAAGGTTAAAGGAAATCACATGATAAGAAGGAGATATACATAT
dgoR-GFP-R	CGCAGATTGGTCGATCCCCAGTCAATTGCGATGTAGCGAGTTATCACTTATTCAGGCGTA
carA-GFP-F	TATCGAGCTTATTGAGCAATACCGTCAGTCCGCGAAATAATAAGAAGGAGATATACATAT
carA-GFP-R2	CTTTTTATATCTGTACGTTTTGGCATGGCTCTTATTACTCCTTATCACTTATTCAGGCGT
ssaN -GFP-F	GCTACTTATAGAAAAATTACACCAAATACTCACCGAGTGATAAGAAGGAGATATACATAT
ssa-GFP-R	TTTTCACGCCGCGCGATTATCTCCAGCAAAGTTTCCATGATTATCACTTATTCAGGCGTA
slrA-GFP- F	ACTTGAGCAGAAAGTTCATCTGACAGGAGCGGTATCATGATAAGAAGGAGATATACATAT
slrA-GFP- R	AGCGGGCCGCCCCAGCCGCCGGCGCCTTTCTCAATGCGAATTATCACTTATTCAGGCGTA
mltB-GFP-F	GCAACTGGGCCAGGCGGTAGCGCTGGCGCGGGTGCGCTAATAAGAAGGAGATATACATAT
mltB-GFP-R	CCCAAAAAGCCATAATAATAGCGGAGGGGGTGCTCCCCTCTTATCACTTATTCAGGCGTA
yihU-GFP-F	GCGCATCAGCGCCGGGCTGACCGCCAACGTAAAAAAGTAATAAGAAGGAGATATACATAT
yihU-GFP-R2	GATGGTGTAGTTGTTCATTCAATTTTCCTTTATACAGTCGTTATCACTTATTCAGGCGTA
STM1290-GFP-Fdel	CCGGTACCAGGCAGCCCTATGCATAAACCTGAAATTGTGGTAAGAAGGAGATATACATAT
STM1290-GFP-R	CTCTGAGGCATAAATATCAATAGTAAGCGTGTTCATTTTCTTATCACTTATTCAGGCGTA
STM5047-GFP-F	CCTTTATGCCTTAGTCAGTAGCTGGCAATACGATTGTTGATAAGAAGGAGATATACATAT

STM5047-GFP-R	GGGTATTATTTTAATTCAACACCGCCGGGCTTTATACCCGTTATCACTTATTCAGGCGTA
STM5308-GFP-F	AAAACTGCAGCCAATTCAAACCGAACGTTATTCTGATTAATAAGAAGGAGATATACATAT
STM5308-GFP-R2	CGATGATCGTATTTAGCGATCATCGGCATTGTGGTGAAACTTATCACTTATTCAGGCGTA
ftnB-GFP-Fdel	GCATCAAATCTTTACCTCCATCTAAGCGAATGGTGTTCTTAAGAAGGAGATATACATATG
ftnB-GFP-R	CTATCCGGCTAATAACGTCTCAATATTGTCGCTCGGGGTGTTATCACTTATTCAGGCGTA
gtrA-GFP-F	CCAGACCTTTCCGAATCCGCTGATTTTCATAATGTTGAAGTAAGAAGGAGATATACATAT
gtrA-GFP-R	GTGGCTTCTTCATTGAAGACCGGAACGACTAACGAGATTTTTATCACTTATTCAGGCGTA
holA-GFP-F	CTGCCATAAGGCGCTGGCAGACGTATTTATTGATGGGTGATAAGAAGGAGATATACATAT
holA-GFP-R	ATCAAAGGTGCCGCCGAATAAAGCCTGTAACGATTTCATATTATCACTTATTCAGGCGTA
STM4889-GFP- Fdel	GTGATTATCGCCATGCAGCTGCTCCTCGCCTATTTCTATTAAGAAGGAGATATACATATG
STM4889-GFP- R	GCCGATAACCCAAACCTTATTCATGGCCTTCATTTGCGCCTTATCACTTATTCAGGCGTA
STM3726-GFP-Fdel	CGTCAGGGAATGGAAGCCAACATCGTACATATTGGATTTTAAGAAGGAGATATACATATG
STM3726-GFP- R	TATTCGATATTTCCCGGATTATTACAGATAAGCGTTTCATTATCACTTATTCAGGCGTA
STM2047-GFP-F	GTTGGGCAGTCGCATGAACACCCTCAGAGAATCGAACTGATAAGAAGGAGATATACATAT
STM2047-GFP-R	CGCTGACGCATGGCGAAAGGATCGCTAAGCGAAACAAATATTATCACTTATTCAGGCGTA
nanA-GFP-F	CAAGGCGGCTGGCTCAACAACTGATGGAAGAAAAGGCGTAATAAGAAGGAGATATACATAT
nanA-GFP-R	ACACTGTTGTAGGCCGGGCAAGCGTAGCGCCCCCGGCATTTTATCACTTATTCAGGCGTA
ssaN-mOrange-F	GCTACTTATAGAAAAATTACACCAAATACTCACCGAGTGATAAGAAGGAGATATACATAT
ssaN-mOrange-R	TTTTCACGCCGCGCGATTATCTCCAGCAAAGTTTCCATGACATATGAATATCCTCCTTAG
gtrA-mcherry-F	CCAGACCTTTCCGAATCCGCTGATTTTCATAATGTTGAAGTAAGAAGGAGTATACATATGGTGAGC

	AAGGGC
gtrA-mcherry-R	GTGGCTTCTTCATTGAAGACCGGAACGACTAACGAGATTTTGAATATCCTCCTTAGTTCC
carA-mOrange-F	TATCGAGCTTATTGAGCAATACCGTAGTCCGCGAAATAATAAGAAGGAGATATACATAT
carA-mOrange-R	CTTTTTATACTGTACGTTTTGGCATGGCTCTTATTACTCCCATATGAATATCCTCCTTA
nanA-gatc-F	GGCACGCGCTCGGTAAAAAA
nanA-gatc-R	GAGGTGCGATCGCTACCGCA
nanA-NO gatc-F	GAAAGCGCTGGAAATTAATA
nanA-NO gatc-R	CAGCCAGCACCCGGTGAAAC
