# Supplementary material for: Contribution of DNA adenine methylation to gene expression heterogeneity in Salmonella enterica
Source: Nucleic Acids Res. 2020 Sep 21;48(21):11857–67. doi: 10.1093/nar/gkaa730 (PMC7708049; doi:10.1093/nar/gkaa730)
Supplement: gkaa730_Supplemental_Files [file gkaa730_supplemental_files.zip › Table S3.pdf]

M. A. Sánchez-Romero, D. R. Olivenza, G. Gutiérrez and J. Casadesús

Table S3. Nonmethylated GATC sites in the genome of *S. enterica* ser. Typhimurium strain ATCC 14028

Genome position of the A (GATC)	Location	DNA strand	Gene/operon function	Position of GATC relative to the transcription start point
75370	*carA*	+	Carbamoyl-phosphate synthase small chain	-511
75675	*carA*			-206
75676	*carA*			
618472	*gtr*	-	O-antigen glycotransferase	-56
618473	*gtr*			
618485	*gtr*			-69
618486	*gtr*			
709012	*holA*	-	DNA polymerase III, delta subunit	+4
1177764	*STM1290*	-	N-acetylmannosamine-6-phosphate-2-epimerase	-465
1177765	*STM1290*			
1290523	Within ORF			
1290524	Within ORF			
1470525	Within ORF			
1505465	*ssaN*	+	Type III secretion ATP synthase	-202
1505466	*ssaN*			
1640964	Within ORF			
1641075	Within ORF			
1661988	Within ORF			
1661989	Within ORF			
1662036	Within ORF			
1670093	Within ORF			

1712903	Within ORF			
1749020	Within ORF			
1800447	*STM2047*	-	Hypothetical protein	-68
1871314	Within ORF			
1893998	Within ORF			
2038305	*ftnB*	+	Ferritin-like protein	-340
2038489	*ftnB*			-156
2361416	*opvAB*	-	Regulation of O-antigen chain length	-105
2361417	*opvAB*			
2361489	*opvAB*			-178
2361490	*opvAB*			
2392046	Within ORF			
2392047	Within ORF			
2467804	Within ORF			
2998457	*slrA*	+	Glucitol/sorbitol-specific enzyme IIC component	-86
3265045	*STM3726*	-	Putative manitol dehydrogenase	-68
3522967	*nanA*	-	N-acetylneuraminate lyase	-58
4047784	*dgoR*	-	Galactonate operon transcriptional repressor	-147
4248310	*yihU*	-	Hypothetical oxidoreductase	-66
4248311	*yihU*			
4289017	*STM4889*	+	Putative Na^+/galactosidase symporter	-171
4289018	*STM4889*			
4433484	*STM5047*	-	Putative cytoplasmic protein	-108
4673845	*STM5308*	-	Sugar transporter	-157
4673846	*STM5308*			
