# Supplementary material for: Contribution of DNA adenine methylation to gene expression heterogeneity in Salmonella enterica
Source: Nucleic Acids Res. 2020 Sep 21;48(21):11857–67. doi: 10.1093/nar/gkaa730 (PMC7708049; doi:10.1093/nar/gkaa730)
Supplement: gkaa730_Supplemental_Files [file gkaa730_supplemental_files.zip › Table S4.pdf]

Table S4. DNA sequence of the promoters and upstream regulatory regions of loci harboring undermethylated GATC sites in the genome of *S. enterica* ser. Typhimurium strain ATCC 14028. GATC motifs (in red) and binding sites for transcription factors are shown. The region from the promoter to the start codon of the ORF (in orange) is also included. The sequences start 20 nucleotides upstream of the signal (GATC or protein binding site) most distant from the promoter.

Gene	Sequence	Transcription factor(s) involved
carA	tgtcttaaagaaaaagaagctggatctttatatcgcgattgtaattcttattacattgtcttaatttgctgttttt<br>tgttgattttatgatgcatttgatttgtttttatatttttaatatattgattttaaaatttttttgtcgttaac<br>tttcttgattttgaatttttatgcatttaatggcattgatggctattaagtgagtttttatcgttgtttttttgttg<br>ttttaatgtcaaatttgaccatttggtccactttttatcatgccagccagtttttttgcgaactcaaggaagcgcaa<br>gcgttttctatcgtaacttagttgatctttttgcctcttaaacagaataacttccttataatgtgcaaaataacat<br>aaaaaacaccctctttaagttgacttttatccggcttaacttcagaatgccgccgtttgccagaaatccacgggta<br>agcaaatttgcattgcttcatactgactgaatgaattaatatgcaaataaagtgagtgaatattctctggagggtg<br>tt**TTG**	CRP
dgoR	cccataaccaattgtcgttttttgtgatctaaattgtagtacaacataattgtgttgtactacattaatggcatgat<br>aacgacggttgatatcacgctagtactacaaaattgcggcgtaattcagctatcgcggtaaagtaagagagttcac<br>atcgagcacaaggactctct**ATG**	CRP
gtr	agcaatcagtagccccaattgATCGGTAACAACGATCaattaataagataacaataactttaaactattgaatacc<br>acattattgATCGTTTATATCGATCaaagcaatttgtagtgctacactccagacctttccgaatccgctgattttc<br>ataATG	OxyR (in capitals)<br>CRP<br>Fur
holA	tccggcttcaatgaaaacgatgatcctcgattccggcgatccgaacggtccgctaagtcgtgcggtacgtaatcag<br>ttacggctgaataacgtaaacctgcttgataaagacacaacgcgcaaagacgttccgtctctgcggcttggaacgg<br>tgactattttgcaggatacggcgtcagtgttccaggacggccagacggcggaatatcagatggtgatgaccgtcaa<br>tgcctcggtattgattccgggccatgacatctatcctatcagcacgaaagtgtaccgttcgttcttcgataacccg<br>cagatggcgttggcgaaggataacgagcaggccatgatcgttcaggagatgtacgacaaagccgctgaacagctga<br>ttcgtaagctgaccagcgtccgcgcggcggatattcaggcgacgaaagaggaggctaccgccgacaatgagacggc<br>ggcgcccgcctctacgccagcacgcgtctccactacgctgagtaactaATG	CRP *<br>Fur *

*nanA***	gaacatgctaccgacgagcagatcgcgttgttaacgaaagcgctggaaattaatagccagtcgctggatgacaacg<br>cgctgtttattcgttcagatgtcgagtttcaccgggtgctggctgaaattcccggcaatccgatctttatggctat<br>tcatgttgctctgctggactggctaatcgccgctcgcccaagcgttcccgatcgtgagctgcatgaacataataac<br>gtgagttatcaacaacatattgtgattgttgatgccattcgccagcgagatcccgataaagcggatcgcgctctgc<br>aaacccacctcaatagcgtttccgctacctggcacgcgctcggtaaaaaaagtcaaaaatgcggtagcgatcgca<br>cctccgatcccggtttagtgaagcagatcgcactataagcgttctgtattatttgctgcttatttgatctggtata<br>acaggtataaaggtaggtagttaatatattcatcatccgtagaggtaggtATG	CRP<br>OxyR
opvAB	ttggctcgcctttacgcccaaaagtgtgctctggtttgcaaccgacaggggacatgtgtttaacgacccgggaaga<br>ggccattatttatttgctggaactcgaacagaaaaccttaccatgagctatgccgtttttgataaacgtgcagggg<br>cttacgttatatcccgaaatagtctgcaaaagctgcgcctcaaattacctccttttaccattaaatagcgtaatac<br>atgattcccttatttcgtggcttctgaagcggtagatgtcgccgctcaaccccgttggtgcctgaacgtgtaccga<br>atgaaccgtcacataaaacaaaaacgatcaattttatttatatgATAGTTTTTATCTATtaaaaagaggaattttg<br>ATCGATTTTAATTATttataacgatcgatatcatgaatttgATAGTTTATATCTATcattgatgtatttaccgATC<br>GATATAACCAGTgtgaatgtattgttattttcctttaaaatactcgaattcttATG	OxyR (in capitals)<br>CRP<br>Fur
ssaN	agagcttcagcgccagttacccattaataaaatcgctgaaactttgcaacggcttgtatcagagcgggtttctatt<br>agagatttacgtcttattttcggcaccttaattgactgggcgccacgtgaaaaagatgtcctgatgttgacagaat<br>atgtccgtatcgcgcttcgtcgtcatattctgcgtcgtcttaatccggaaggaaaaccgctgccgattttgcggat<br>cggcgaaggtattgaaaacctcgtgcgtgaatccattcgccagacggcaatggggacctatactgcgctgtcgtct<br>cgtcataagacgcagatcctgcaacttatcgagcaggcgctgaagcagtcagccaaattattcattgtcacttctg<br>tcgacacccgacgtttcttgcgaaaaattacagaagccaccttgttcgacgtaccgattttgtcatggcaggaatt<br>aggagaggagagccttatacaagtggtagaaagtattgaccttagcgaagaggagttggcggaca**ATG**	CRP<br>OxyR
STM1290	agtaaagcttcatcattagtgatcttatggtgcacaatttcaacaatgacaatgacaaaatgtgaccaagcgctaa<br>aaataattacatggagttatcatgtgcgaggtaaaagcaccccatggtgatgttagaagtgattatgggtgatctg<br>ttatggccaaactcattagtgatagcattctgaataaatacaacaagaattatgtgatgcatttcatactggctat<br>cccgtggcgtataagctccttctgagcattttatctttatgccggaagcagttaataaaatacagcatgctgccgt<br>cagaatgaattgtatttgtcttgtagcttcaggttatagcctcaaatccgctggcgcagtaattgaataacaaaca<br>taccttatgactgtgttcacatttttctatctttttgcttgatgtagcaacactccaagacataacataaaacgga<br>gcaaaacttcaaatatataaggcggaactgg**ATG**	CRP

* Binding sites not found

** A GATC site at position +4 is not shown
